# Microbial environment shapes immune function and cloacal microbiota dynamics in zebra finches *Taeniopygia guttata*

**DOI:** 10.1186/s42523-020-00039-3

**Published:** 2020-06-03

**Authors:** H. Pieter J. van Veelen, Joana Falcão Salles, Kevin D. Matson, Marco van der Velde, B. Irene Tieleman

**Affiliations:** 1 Groningen Institute for Evolutionary Life Sciences, University of Groningen, P.O. Box 11103, 9700 CC Groningen, The Netherlands; 2 Wetsus, European Centre of Excellence for Sustainable Water Technology, Oostergoweg 9, 9811 MA Leeuwarden, The Netherlands; 3 Resource Ecology Group, Department of Environmental Sciences, Wageningen University, P.O. Box 47, 6700 AA Wageningen, The Netherlands

**Keywords:** Ecological immunology, Microbial environment, Host-microbiota interactions, Microbiota dynamics, Avian microbiota

## Abstract

**Background:**

The relevance of the host microbiota to host ecology and evolution is well acknowledged. However, the effect of the microbial environment on host immune function and host microbiota dynamics is understudied in terrestrial vertebrates. Using a novel experimental approach centered on the manipulation of the microbial environment of zebra finches *Taeniopygia guttata*, we carried out a study to investigate effects of the host’s microbial environment on: 1) constitutive immune function, 2) the resilience of the host cloacal microbiota; and 3) the degree to which immune function and host microbiota covary in microbial environments that differ in diversity.

**Results:**

We explored immune indices (hemagglutination, hemolysis, IgY levels and haptoglobin concentration) and host-associated microbiota (diversity and composition) in birds exposed to two experimental microbial environments differing in microbial diversity. According to our expectations, exposure to experimental microbial environments led to differences related to specific antibodies: IgY levels were elevated in the high diversity treatment, whereas we found no effects for the other immune indices. Furthermore, according to predictions, we found significantly increased richness of dominant OTUs for cloacal microbiota of birds of the high diversity compared with the low diversity group. In addition, cloacal microbiota of individual females approached their baseline state sooner in the low diversity environment than females in the high diversity environment. This result supported a direct phenotypically plastic response of host microbiota, and suggests that its resilience depends on environmental microbial diversity. Finally, immune indices and cloacal microbiota composition tend to covary within treatment groups, while at the same time, individuals exhibited consistent differences of immune indices and microbiota characteristics.

**Conclusion:**

We show that microbes in the surroundings of terrestrial vertebrates can influence immune function and host-associated microbiota dynamics over relatively short time scales. We suggest that covariation between immune indices and cloacal microbiota, in addition to large and consistent differences among individuals, provides potential for evolutionary adaptation. Ultimately, our study highlights that linking environmental and host microbiotas may help unravelling immunological variation within and potentially among species, and together these efforts will advance the integration of microbial ecology and ecological immunology.

## Background

Diverse microbial communities are ubiquitous components of animals and the aquatic and terrestrial ecosystems that they inhabit [1]. The immune systems of animals invariably deal with numerous microbial organisms at any given place and time, and have consequently evolved to prevent microbial over-exploitation, infection and disease (i.e. parasitism) and to allow beneficial (i.e. mutualism) and neutral host-microbe interactions (i.e. commensalism). Studies in a relatively new research domain, ecological immunology, have begun to reveal some sources of immunological variation across species [2–6], among individuals [7–9], and during life cycles [10, 11]. However, a large part of this work has collectively demonstrated that immunological variation is poorly aligned with life history strategies among species (e.g. pace-of-life) [e.g. 5, 6]. Likewise, immunological variation within individuals frequently does not follow predictions based on life-history trade-offs [7, 11, 12]. Instead, immunological variation often is better correlated with environmental variability [3, 9, 13, 14], supporting ideas that animals optimize immune defenses to fit their environment, on both evolutionary and ecological time scales [14–16]. The pathogenic and nonpathogenic effects of microbial life on wildlife health and fitness and the origins, maintenance, and disturbance of animal-microbe interactions represent major frontiers in contemporary biology [17–19]. One important unresolved issue is whether the environmental microbial communities encountered by an animal affect the immune function, and ultimately survival, of that animal [15, 16].

Another component of the interface between a host and its environment is the host-associated microbiota, the sum of the microbial communities residing in and on an animal’s body. Like immune function, host-associated microbiotas show tremendous variation among species and individuals and through time and space [20–24]. The status of host-associated microbiotas is currently debated: some view the host-associated microbiota as a phenotypic trait of its host; others see the microbiota and the host as a meta-organism [25–28]. Regardless, several fundamental questions in this debate remain to be addressed, including whether the host-associated microbiota is determined by inheritance or by the environment, and whether the host’s microbiota acts as a phenotypically plastic trait for quickly responding to versatile environments [15, 29]. Understanding the latter requires concomitant measurement of host-associated and environmental microbial communities; however, this type of work is just beginning to be carried out in terrestrial nonhuman vertebrates. Irrespective of whether the microbiota should be defined as a host trait or not, the conceptual distinction between an animal’s microbiota and its (microbial) environment fades as a result of weak host-microbe partner fidelity [28], common host-environment microbial exchange [30, 31], or both. Ideally, testing effects of the microbial environment on host-associated microbiota diversity, composition and dynamics should be done while controlling for factors known to shape animal microbiota [29, 32–36], such as diet or sex [37, 38].

Individual animals routinely experience very different environments within their lifetimes, for example when migrating or when seasons change [reviewed in [39]. As a prerequisite for investigating how microbial environments shape host immunological phenotypes via host-associated microbiota, quantifying the resilience of host-associated microbiota to shifts in environmental microbial communities may prove vital. Tracking how the host-associated microbiotas of individuals respond to novel microbial environments [e.g. 40] will offer insights into the individuality, flexibility and resilience of microbiota traits, and into the time span at which responses to novel microbial environments occur. Earlier attempts at this type of tracking did not control for important confounding factors, e.g., dietary effects on gut microbiota variation [41, 42]. Hence, experimental approaches that subject animals to novel microbial environments while limiting confounding effects are needed, and need also consider the individuality of responses. Widely used indices of immune function can fluctuate temporally within individuals; simultaneously, individuals can consistently differ, i.e., be repeatable [43, 44]. Host-associated microbiota can similarly show signs of individuality but see [45, 46]. Accordingly, questions about individual-level connections between host immune function and host-associated microbiota have emerged [15, 16], and call for simultaneous assessment of immune function and host-associated microbiota.

While not investigated in an ecological immunology framework, studies of constitutive immunity in humans and rodent models implicated that levels of specific antibodies [47, 48], polyclonal natural antibodies [49], and complement activity [50] were positively associated with gut microbiota diversity. Here, we describe an experiment in which we manipulated the microbial environment to test its influence on innate and adaptive aspects of immune function and on the diversity and resilience of host-associated microbiota of captive zebra finches *Taeniopygia guttata*. 1) We explored temporal patterns of immunity and cloacal microbiota characteristics over 8 weeks in birds that were continuously exposed to one of two experimental environments that differed in microbial diversity and composition. Based on the literature, we predicted that, if constitutive levels of antigen-specific IgY, natural antibodies and complement-like factors are influenced by the diversity of environmental microbial communities, their concentration would increase in response to high environmental microbial diversity. In addition, if infection incidence increases with microbial diversity, we predicted elevated levels of haptoglobin, a marker of inflammation [44], under high environmental microbial diversity. We accordingly predicted decreasing or a lack of patterns under conditions with low environmental microbial diversity. 2) We also investigated whether microbial environments with different diversities affected the diversity and resilience (i.e. degree and time to recovery) of the cloacal microbiota. We minimized dietary influences on the microbiota by supplying sterilized food and water. We then predicted that a more diverse microbial environment would increase the diversity and slow the recovery of cloacal microbiota. 3) Finally, we examined correlations between immune indices and host-associated microbiota characteristics, where correlations would suggest that vertebrate immune function responds to environmental microbiota within 8 weeks. Our longitudinal study design additionally allowed us to quantify repeatability of immune indices and host-associated microbiota characteristics.

## Results

### Microbial environment affects IgY concentration but not innate immune indices

To experimentally test if microbial environments (Additional file 1: Fig. 1) affect indices of immunity, we moved 53 adult females and 54 adult males from single-sex outdoor aviaries to indoor cages (50 X 50X 40 cm), each of which housed two birds of the same sex. We supplied all cages with bedding materials comprising soils with bacterial communities of high (Shannon *H′* ± SE = 5.6 ± 0.05) or low bacterial diversity (3.9 ± 0.05) and different community compositions (Additional file 1: Fig. 1). Each of the two replicate rooms per experimental microbial environment contained 12 cages arranged in a 3 X 4 grid with alternating male and female cages. Birds were randomly assigned to a room and a sex-specific cage (see Additional file 1 for more details on experimental procedure and housing conditions). We provided a standardized diet of ad libitum gamma-irradiated seed mixture and autoclave-sterilized water to all birds. The water was supplemented with 4 g l^− 1^ of a micropore-filtered multivitamin-amino acid solution (Omni-vit, Oropharma N.V., Deinz, Belgium) to compensate potential irradiation-induced vitamin degradation in seed. We measured indices of innate (agglutination titer of natural antibodies, complement-mediated lysis titer, and haptoglobin concentration [44, 51]) and adaptive immune function (total plasma concentration of immunoglobulin Y (IgY), i.e. the avian equivalent of IgG [52, 53]), in females at four time points: < 1 day before the experiment (i.e. baseline) and after weeks 2, 4 and 8 of the experiment. We analyzed only females because of practical limitations, and cloacal swabbing was impossible for males. We evaluated time effects using four distinct sampling days, which we considered categorically in order to determine within-individual changes between these sampling moments.

**Fig. 1:**
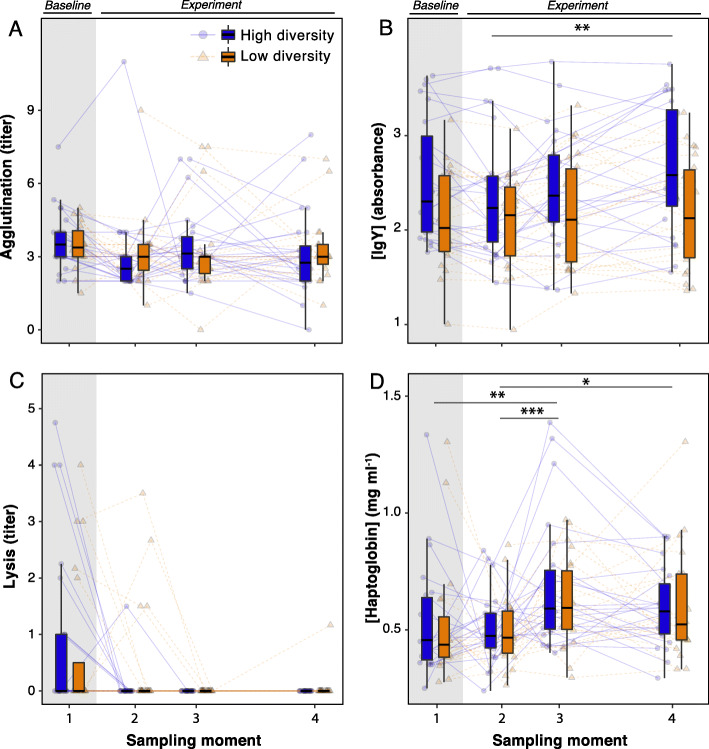
Experimental and temporal effects on host immune function. Relationships of population-level variation of (**a**) agglutination titer, (**b**) IgY concentration, (**c**) lysis titer and (**d**) haptoglobin concentration across sampling moments, stratified by experimental treatment. Faded blue circles (high diversity soil) and orange triangles (low diversity soil) represent individual measurements connected by a line per individual female (solid = high diversity, dashed = low diversity). Boxplots show median and first and third quartile per group, with whiskers representing 1.5 · IQR. Treatment groups were measured simultaneously but split along x-axis for visual clarity. Grey area highlights the baseline sampling moment. Experimental treatment and temporal effects on lysis titer were analyzed as occurrence of lytic activity. Asterisks above plots denote pairwise contrasts among sampling moments; * FDR-corrected *q* < 0.1, ** *q* < 0.01. Statistics are detailed in Table 1. The experimental effect on IgY concentration is also significant after exclusion of baseline samples (*F* = 4.35, *P* < 0.05)

Comparing treatment groups, IgY concentration was significantly elevated in the high diversity compared with the low diversity microbial environment (Fig. 1b). This pattern remained when baseline values were excluded (*F*_1, 44_ = 4.35, *P* = 0.04), which we tested separately as baseline values differed between treatment groups despite randomized allocation to treatments (*χ*^2^ = 4.21, df = 1, *P* = 0.04). Agglutination titer, lysis titer and haptoglobin concentration were unaffected (Fig. 1a, c and d; Table 1). The effect on IgY was most strongly present after eight weeks of exposure to the different experimental microbial conditions (Fig. 1b, Table 1). Using a multivariate distance-based redundancy analysis of the four immune indices combined we found no significant difference between treatment groups (*F*_1, 39–43_ < 1.20, *P* > 0.26). The elevated IgY levels in the high diversity microbial environment suggest that antigen-specific antibodies had increased with environmental microbial diversity, whereas agglutination, which is driven primarily by polymeric natural antibodies (e.g. IgM) with low specificity and low affinity, was not different between high and low diversity microbial environments.

**Table 1:** Statistics of longitudinal analysis of experimental and temporal effects on innate immune function

Response	Predictor	Df ^a^	*F*	*P*
Agglutination (titer)	Experimental treatment	1, 41	0.03	0.87
	Sampling moment	3, 125	0.79	0.50
	Interaction	3, 125	0.22	0.88
[IgY] (absorbance)	Experimental treatment	1, 44	5.15	**0.028**
	Sampling moment	3, 129	4.12	**0.008**
	Interaction	3, 129	1.60	0.19
			*z*	*P*
Lytic activity (probability) ^b^	Experimental treatment	1	−0.71	0.48
	Time (days)	1	−2.61	**0.009**
	Interaction	1	1.80	0.07
			*F*	*P*
[Haptoglobin] (mg ml^−1^)	Experimental treatment	1, 44	0.19	0.66
	Sampling moment	3, 127	6.20	**< 0.001**
	Interaction	3, 127	0.40	0.76

^a^Denominator degrees of freedom based on Satterthwaite approximation

^b^No detected lysis titers at sampling moment 3 and 4 inhibited evaluation of differences among sampling moment categories; a logit link GLMM with continuous temporal predictor was fitted instead

We examined temporal shifts in the immune indices to determine if microbial environments altered host immune function. Absence of significant treatment by sampling moment-interactions indicated that changes in immune function between sampling moments were largely independent from experimental microbial conditions (Fig. 1; Table 1). Specifically, while agglutination titers showed no differences between sampling moments at all (Fig. 1a; Table 1), total antigen-specific IgY concentrations increased by 0.19 absorbance units between sampling moments 2 and 4 (*χ*^*2*^ = 12.16, FDR *q* = 0.003; Fig. 1b), and haptoglobin concentration increased by 0.16 mg ml^− 1^ between sampling moments 2 and 3 (Fig. 1d). We observed complement-mediated lytic activity in only a few individuals at the baseline measurement, and the probability of lytic activity further declined after exposure to experimental conditions (Fig. 1c; Table 1). IgY concentrations tended to increase during the experiment only in birds exposed to the high diversity microbial environment (Fig. 1b), but the interaction between treatment and sampling moment was not significant (Table 1), also when baseline measures were excluded (*F*_2, 87_ = 1.53, *P* = 0.22).

To examine the amount of variance in immune indices explained by differences among individuals, we examined the repeated measures on individuals over time, following [54], and revealed that immune function differed consistently among individuals (Fig. 1; Table 1). The repeatability was highest for IgY concentration, and repeatabilities for agglutination titer and haptoglobin concentration were lower, but still significant (Table 2).

**Table 2:** Repeatability of innate immune indices and cloacal microbiota characteristics of female zebra finches

Immune index	Treatment	Principal coordinate axis	*R*	SE	95% CI (lower, upper)^b^	*P*^c^
Agglutination (titer)			0.14	0.07	0.017, 0.301	**0.033**
[IgY] (absorbance)			0.80	0.05	0.65, 0.866	**0.001**
Lytic activity (probability)^a^			0.06	0.20	0, 0.894	0.198
[Haptoglobin] (mg ml^−1^)			0.26	0.08	0.113, 0.423	**0.001**
Multivariate immune function	High diversity	axis 1	0.37	0.13	0.082, 0.584	**0.004**
		axis 2	0.56	0.13	0.265, 0.75	**0.001**
	Low diversity	axis 1	0.08	0.11	0, 0.333	0.242
		axis 2	0.74	0.11	0.437, 0.85	**0.001**
*Cloacal microbiota*						
OTU richness			0.18	0.10	0.005, 0.394	**0.019**
Shannon’s diversity			0.23	0.09	0.064, 0.421	**0.005**
Cloacal taxon occurrence (unweighted UniFrac)	High diversity	axis 1	0.00	0.07	0, 0.223	1.000
		axis 2	0.46	0.13	0.168, 0.662	**0.001**
	Low diversity	axis 1	0.00	0.00	0, 0	0.980
		axis 2	0.28	0.14	0, 0.536	**0.008**

^a^No detected lysis titres at sampling moment 3 and 4 inhibited evaluation of differences among sampling moment categories; a logit link GLMM with continuous temporal predictor was fitted instead

^b^Confidence intervals based on 1000 parametric bootstraps

^c^*P*-values calculated based on 1000 permutations

### Microbial environment affects host-associated microbiota structure and composition

To investigate the diversity and resilience of host-associated microbiota traits in response to different microbial environments, we characterized the host-associated microbiota using cloacal swabs that were collected at the same four time points described above. We extracted DNA from these swabs and characterized the host-associated microbiota through 16S rRNA gene amplicon sequencing (V4/V5 region) using Illumina Miseq (see Additional file 1 for more detail on bioinformatics procedures). Briefly, we assembled quality-filtered sequences into operational taxonomic units (OTUs; 97% ID; see Additional file 2) to analyze alpha and beta diversity. Rarefaction curves indicated that Shannon diversity but not OTU richness reached a plateau, which implied that our sequencing effort was insufficient to document rare OTUs (Additional file 1: Fig. 2). Accordingly, we interpreted OTU richness as the richness of dominant OTUs. Our dataset contained 1,084,107 quality-filtered reads clustered in 1393 OTUs (each contributing > 0.001% of total abundance). Of these OTUs, 81% were shared between the treatments (Additional file 1: Fig. 3), and 168 and 97 OTUs were detected only in birds on high diversity and low diversity soils, respectively. To evaluate host-associated microbiota alpha diversity, we rarefied host-associated microbiota data to 1273 reads per sample (i.e. upper 80% of coverage distribution) for comparability: 173855 reads binned in 1310 OTUs. Beta diversity was calculated based on a non-rarefied and variance-stabilized community table (see *Methods*).

**Fig. 2:**
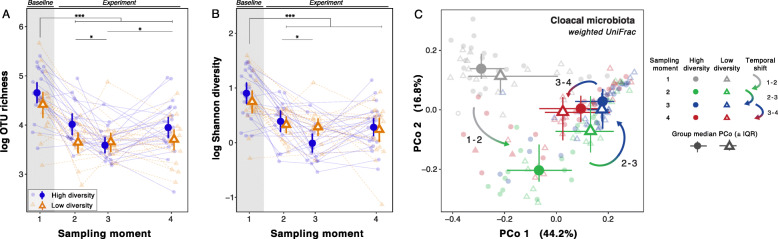
Experimental and temporal effects on cloacal microbiota alpha diversity and phylogenetic beta diversity. Relationships of population-level variation (mean ± 95% CI whiskers) of (**a**) dominant OTU richness and (**b**) Shannon diversity for each experimental treatment and across sampling moments. **c** PCoA of weighted UniFrac distances among cloacal microbiota samples; ordination of all samples including baseline samples shows differential clustering of experimental treatment (closed circle = high diversity, open triangle = low diversity) and sampling moments (colors), as well as a pattern of transitions (bicolored arrows) that first diverges from and later converges toward the baseline state. Group medians and IQR are shown as large symbols and whiskers. **a**, **b** Faded blue closed circles (high diversity) and orange open triangles (low diversity) represent individual measurements connected by a line per individual (faded solid blue = high diversity, faded dashed orange = low diversity). Experimental treatments are taken simultaneously but split along x-axis for visual clarity. Grey area highlights the baseline sampling moment. Asterisks above plots denote pairwise contrasts among sampling moments; *P* or FDR-corrected *q* < 0.1 *, 0.01 **, 0.001 ***. Statistics are detailed in Tables 3 and 4

The experimental microbial conditions led to modest differences in alpha (Fig. 2a and b) and beta diversity of host-associated microbiota (Fig. 2c). Linear mixed model (LMM) analyses of alpha diversity (OTU richness and Shannon diversity) revealed significantly higher richness of dominant OTUs in the host-associated microbiota of birds living on high diversity soils compared with low diversity soils (Fig. 2a, Table 3). We found no significant effect of microbial environment on Shannon diversity of host-associated microbiota (Fig. 2b*,* Table 3). Principal coordinates analysis (PCoA) of weighted UniFrac distances revealed that the phylogenetic composition of host-associated microbiota differed significantly but modestly (1.9%) between experimental groups (PERMANOVA) (Fig. 2c, Table 3). We observed that the composition of pre-experiment samples was more distinct from later sampling moments during exposure to experimental microbial environments (i.e. 2 to 4) (Fig. 2c, Table 3). The relative abundance of major taxonomic groups in the cloacal microbiota of both experimental groups showed similar patterns, with Epsilonproteobacteria, Firmicutes and Actinobacteria representing the most abundant groups once under experimental conditions (Additional file 1: Fig. 4). Transformed OTU counts were modelled with a *DESeq2* [55] negative-binomial generalized linear model (GLMs) with treatment and sampling moment as terms, which did not identify differentially abundant taxa between birds on high and low diversity microbial environments at OTU-level (FDR-corrected *q* > 0.1).

**Table 3:** Statistical analysis of host-associated microbiota alpha diversity

*Response*	*Predictor*	df ^a^	*F*	*P*
OTU richness (log-scale)	Experimental treatment	1, 43	4.56	**0.04**
	Sampling moment	3, 104	35.01	**< 0.001**
	interaction	3, 104	1.42	0.24
			*F*	*P*
Shannon’s diversity (log-scale)	Experimental treatment	1, 42	0.00	0.99
	Sampling moment	3, 103	28.35	**< 0.001**
	interaction	3, 103	2.43	0.07
Δ OTU richness (y_t_ - y_t-1_)	Experimental treatment	1, 76	0.75	0.39
	Sampling interval	2, 76	22.42	**< 0.001**
	interaction	2, 76	2.56	0.08
Δ Shannon’s diversity (y_t_ - y_t-1_)	Experimental treatment	1, 76	0.80	0.37
	Sampling interval	2, 76	17.37	**< 0.001**
	interaction	2, 76	1.98	0.14

^a^Denominator degrees of freedom based on Satterthwaite approximation

To address the resilience of host-associated microbiota in response to the novel environments, we evaluated the change in host-associated microbiota characteristics from outdoor aviary conditions to the indoor experimental treatments (at sampling moment 2). We found that alpha diversity declined (Fig. 2a and b) and beta-diversity shifted in both treatment groups (Fig. 2c; Table 3). Non-significant interactions between treatment and sampling moment indicated that these compositional changes were independent of the experimental microbial conditions (Table 3; Table 4). *DESeq2* analysis revealed that normalized OTU abundance changes were largely caused by a (near) complete loss of some bacterial phyla after first exposure to experimental microbial conditions (e.g. loss of Bacteroidetes, Cyanobacteria and Fusobacteria). Subsequent analysis of changes of OTU abundances in the host-associated microbiota during the experiment (between sampling moments 2 and 4) revealed abundance changes that were inferior to those induced by outdoor-to-indoor translocation of birds (Additional file 1: Figs. 4 and 5). Shifts were most evident for Proteobacteria classes, where Epsilonproteobacteria, which were not dominant in soils (Additional file 1: Fig. 1e), became relatively more dominant in host-associated microbiota at the expense of Alpha- and Betaproteobacteria (Additional file 1: Fig. 4). The detection of Chloroflexi, Chlamydiae and Firmicutes in host-associated microbiota was clearly associated with acclimation to experimental conditions irrespective of treatment group (Additional file 1: Fig. 1e). At the OTU level, nine taxa assigned to genus *Lactobacillus* (*n* = 5), genus *Campylobacter* (*n* = 2), family *Enterobacteriaceae* (*n* = 1), and family *Micrococcaceae* (*n* = 1) significantly changed in abundance with experimental duration (Table 5), but none of these responses were treatment-dependent (FDR-corrected *q* > 0.1).

**Table 4:** Adonis2 and linear mixed model statistics of experimental and temporal effects on phylogenetic beta diversity

**Phylogenetic beta diversity**				
*Adonis(2)*^*a*^	R^2^ (%)	df	Pseudo-*F*	*P*
Experimental treatment	1.86	1	3.87	**0.01**
Sampling moment	26.87	3	19.73	**< 0.001**
interaction	2.21	3	1.64	0.08
**Within-individual weighted UniFrac distance (y**_***t***_*-***y**_***t-1***_**)**				
*ANOVA*		df	*F*	*P*
Experimental treatment		1, 95	3.51	0.06
Sampling interval		2, 95	17.05	**< 0.001**
interaction		2, 95	5.28	**< 0.01**
*Contrasts* (sampling interval)	effect	Df	*χ*^*2*^	FDR *q*
High - Low diversity (1–2)	−0.02	1	0.14	0.87
High - Low diversity (2–3)	0.17	1	13.21	**< 0.001**
High - Low diversity (3–4)	− 0.01	1	0.03	0.87

^a^Group dispersions are shown in Additional file 1: Fig. 8

**Table 5:** Log2 fold change and taxonomic affiliation of temporally changing OTUs in cloacal microbiota

Log2 fold change with experimental duration per day					
OTU ID	Mean of normalized counts	log2FoldChange	SE	Wald statistic	FDR *q*
OTU828667	1.26	0.07	0.012	5.71	0.000
OTU221299	4.06	−0.02	0.006	−3.79	0.018
OTU1107027	0.78	−0.05	0.013	−3.67	0.019
OTU955052	2.40	−0.03	0.008	−3.57	0.021
OTU333178	0.53	−0.04	0.015	−2.91	0.094
New.ReferenceOTU128	1.17	−0.04	0.012	−3.23	0.044
OTU922761	0.44	0.06	0.019	3.22	0.044
New.ReferenceOTU261	1.95	0.02	0.006	3.33	0.041
New.ReferenceOTU434	1.17	0.03	0.009	3.03	0.072
Taxonomic affiliation					
OTU ID	Phylum	Class	Order	Family	Genus
OTU828667	Actinobacteria	Actinobacteria	Actinomycetales	*Micrococcaceae*	unassigned
OTU221299	Firmicutes	Bacilli	Lactobacillales	*Lactobacillaceae*	*Lactobacillus*
OTU1107027	Firmicutes	Bacilli	Lactobacillales	*Lactobacillaceae*	*Lactobacillus*
OTU955052	Firmicutes	Bacilli	Lactobacillales	*Lactobacillaceae*	*Lactobacillus*
OTU333178	Firmicutes	Bacilli	Lactobacillales	*Lactobacillaceae*	*Lactobacillus*
New.ReferenceOTU128	Firmicutes	Bacilli	Lactobacillales	*Lactobacillaceae*	*Lactobacillus*
OTU922761	Proteobacteria	Gammaproteobacteria	Enterobacteriales	*Enterobacteriaceae*	unassigned
New.ReferenceOTU261	Proteobacteria	Epsilonproteobacteria	Campylobacterales	*Campylobacteraceae*	*Campylobacter*
New.ReferenceOTU434	Proteobacteria	Epsilonproteobacteria	Campylobacterales	*Campylobacteraceae*	*Campylobacter*

**Fig. 3:**
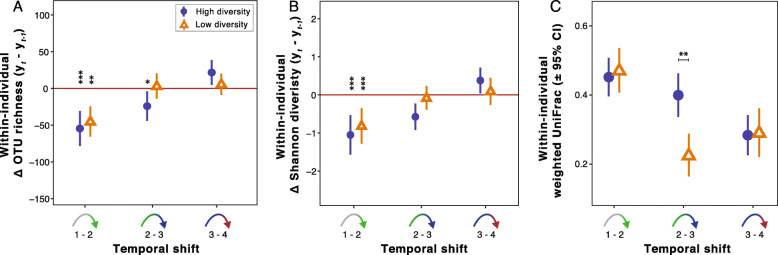
Temporal shifts in host-associated microbiota characteristics across experimental treatment and sampling moments. Average within-individual differences (± 95% CI whiskers) of (**a**) OTU richness, (**b**) Shannon’s diversity and (**c**) weighted UniFrac distance between consecutive sampling moments, presented for each temporal shift (bicolored arrows) and stratified by experimental treatment (closed circle = high diversity, open triangle = low diversity). Associated statistics are detailed in Tables 3 and 4

To address the resilience of host-associated microbiota in different experimental microbial environments, we analyzed within-individual changes in alpha and beta diversity between consecutive sampling moments, and then tested the experimental effect on these temporal shifts. The decline in OTU richness of host-associated microbiota stopped earlier in low than in high diversity experimental microbial conditions (Fig. 3a). Shannon diversity showed a similar pattern but this was not significant (*χ*^*2*^ = 2.61, FDR *q* = 0.32) (Fig. 3b). Moreover, after host-associated microbiota composition moved away from the baseline composition, temporal patterns indicated that compositions returned in the direction of the baseline (Fig. 3c): the composition at sampling moment 4 was more similar to the baseline than to the composition at sampling moment 2 or 3 (*F*_1, 5034_ > 6.47, *P* <  0.016; Additional file 1: Fig. 6). Furthermore, the shift away from the baseline was stronger in birds in the high diversity than in the low diversity microbial environment (Fig. 2c; Additional file 1: Fig. 6). Similar to OTU richness, a within-individual analysis of changes of phylogenetic composition between consecutive sampling moments revealed that host-associated microbiota indeed stabilized earlier in the low diversity microbial conditions (i.e. higher turnover; Fig. 3c; Table 3; Additional file 1: Fig. 7). In addition to the phenotypically plastic responses to environmental microbial conditions, analysis of within-individual repeatabilities of host-associated microbiota alpha and beta diversity indices demonstrated that OTU richness, Shannon diversity, and the second unweighted UniFrac PCoA axis were significantly repeatable (Table 2), suggesting that host-related factors also shaped the host-associated microbiota.

### Immune function and host-associated microbiota correlate at the individual level

**Fig. 4:**
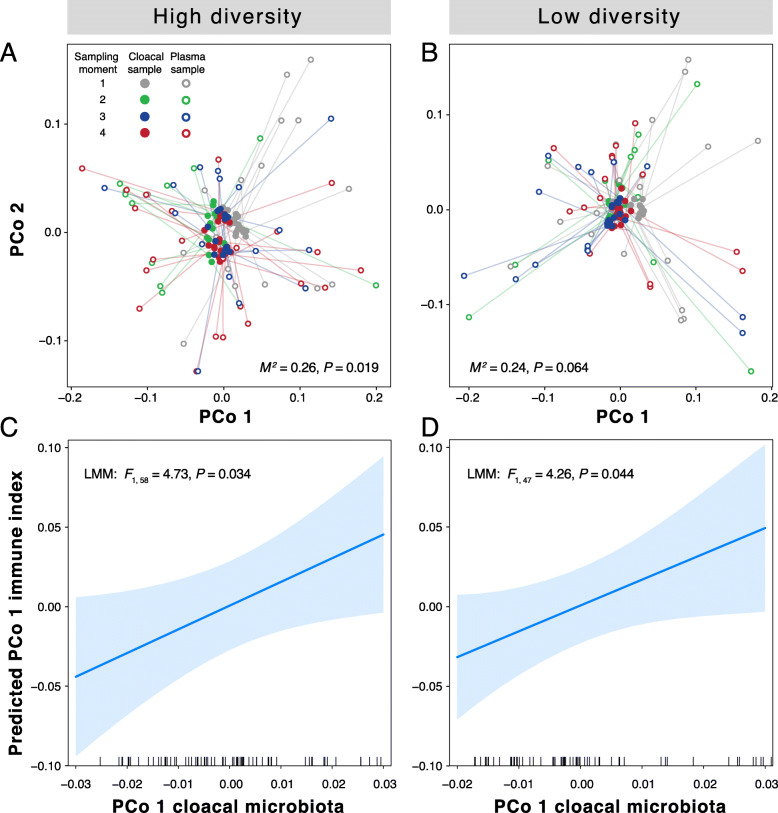
Procrustes analysis of immune function and cloacal microbiota states. **a**, **b** Procrustean superimposition of two multivariate data sets for birds exposed to (**a**) high diversity and (**b**) low diversity soils: multivariate immune index based on four immune indices (agglutination titer, IgY concentration, lysis titer, haptoglobin concentration) (open symbol) and taxon occurrence in cloacal microbiota based on unweighted UniFrac (closed symbol). Procrustes analysis scaled and rotated both ordinations to the best Procrustean fit (*M*^*2*^) and protest statistics are shown in each plot. **c**, **d** PCoA scores of immune function of birds exposed to (**c**) high diversity and (**d**) low diversity soils predicted by PCoA scores for phylogenetic taxon occurrence of cloacal microbiota. The line depicts the predicted relationship and the shaded area depicts the 95% CI of the predictions. **c**, **d** Linear mixed-model inferences are controlled using subject identity and replicate room as random effects

**Fig. 5:**
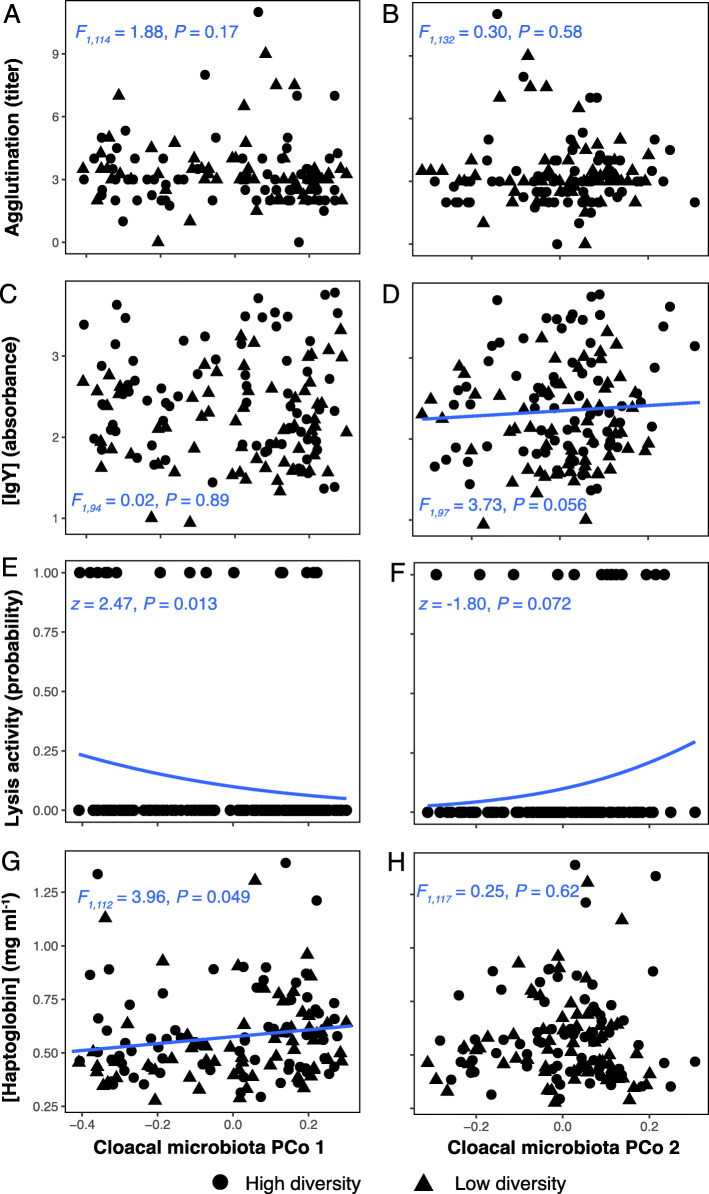
Relationships between individual immune indices and cloacal microbiota PCoA scores. Model predictions (mean = blue line) for (**a**, **b**) Agglutination titer, (**c**, **d**) IgY concentration, (**e**, **f**) Lytic activity and (**g**, **h**) haptoglobin along the first (PCo 1) and second (PCo 2) axis of unweighted UniFrac, respectively. Black dots are individual plasma samples. **g** LMM statistics are shown in each plot

Given consistent individual differences of immune indices and host-associated microbiota traits (Table 2), we asked whether immune function and the host-associated microbiota covaried at the individual level. To examine these relationships, we performed Procrustes ordination analysis, which revealed that the dissimilarity matrix based on the immune indices (hereafter “multivariate immune index”) correlated with the unweighted UniFrac distance matrix representing taxon occurrence in host-associated microbiota (Fig. 4a and b), with (nearly) statistical support for both the high diversity (*M*^*2*^ = 0.26, *P* = 0.02) and low diversity microbial environments (*M*^*2*^ = 0.24, *P* = 0.06). In contrast, we found no significant correlations between immune function and host-associated microbiota structure based on weighted UniFrac (high diversity: Procrustes *M*^*2*^ = 0.18, *P* = 0.33; low diversity: *M*^*2*^ = 0.18, *P* = 0.23). Furthermore, for each experimental group, LMMs (that included individual identity and replicate room as random effects) resulted in significantly positive correlations between the PCo 1 scores for immune function and the PCo 1 scores for taxon occurrence in host-associated microbiota (unweighted UniFrac; Fig. 4c and d). These models also revealed repeatability of the multivariate immune index and taxon occurrence in host-associated microbiota PCo scores along the first and second axes (unweighted UniFrac, Table 2). We also used LMMs to examine relationships between each separate immune index and OTU richness and Shannon diversity of the host-associated microbiota. Neither OTU richness nor Shannon’s diversity accounted for significant variation in any of the individual immune indices (all LMM fixed effects: *P* > 0.11; Additional file 1: Fig. 9). In contrast, PCo 1 scores of taxon occurrence in host-associated microbiota (unweighted UniFrac) were negatively associated with the probability of lytic activity (Fig. 5e) and positively with haptoglobin concentration (Fig. 5g). Microbiota PCo 2 scores positively associated with both IgY concentration (Fig. 5d) and the probability of lytic activity (Fig. 5f), but neither relationship was significant. Both PCo axes were unrelated to agglutination (Fig. 5a and b).

## Discussion

Exposure to distinct experimental microbial environments led to differences in adaptive immune function and in the composition, richness and dynamics of the cloacal microbiota in zebra finches. Importantly, at the individual level, immune function and the cloacal bacterial taxon occurrence covaried significantly, while individuals differed consistently for both immunological and microbiota variables. Indices of immune function changed over the time course of the experiment, but the temporal patterns were not different between experimental microbial environments. In contrast, the manipulated microbial environments did impact alpha and beta diversity, and cloacal microbiota resilience: the microbiota of zebra finches exposed to the low diversity microbial environment stabilized sooner, and microbiota returned in the direction of the baseline compositional state while maintaining individual differences. In the context of ecological immunology, our results suggest that adaptive immune function plastically responds to microbial communities in the surrounding environment, and that innate and adaptive immune function collectively correlate with host-associated microbiota variation at the level of the individual. With the inherent complexity of microbial communities in the wider environment, its impact on the physiological condition and evolutionary fitness of animals is likely more complex vis-à-vis classic ecological interactions like parasitism. A more thorough understanding of the impact of environmental microbes on animal immunity requires a better picture of within-individual flexibility of immune function and the host-associated microbiota.

The premise that environmental microbial communities may determine the immune defenses of animals underlies the increasing integration of microbial ecology research into ecological immunology [1, 15, 16, 19]. We hypothesized that animals may flexibly adjust immune defenses to the microbial environment at a given place and time. Our results suggest that different microbial environments can affect acquired antibody levels (IgY concentration) in captive zebra finches (Fig. 1). Caution is warranted for drawing firm conclusions, as IgY concentration slightly differed between the two experimental groups at baseline. Given the substantial differences among individuals, longer time series and larger sample sizes could help to affirm the observed pattern. The lack of distinction in agglutination titers in the face of different microbial environments is consistent with the unimportance of exogenous antibody stimulation to the production of natural antibodies [56]. This highlights that differences in the antigenic universe (sensu [16]), here as result of different environmental microbial communities, do not affect all immune defenses equally. Complement-like lysis was low in our zebra finches. This could be a feature of zebra finches [51]. The observed lack of experimental treatment effect corresponds with earlier findings of lysis titers in zebra finches that did not change after manipulation of nest bacterial loads [57]. The concentration of the acute phase protein haptoglobin signals inflammatory status [44, 58]. Accordingly, the lack of any experimental effect on haptoglobin concentration suggests that the experimental microbial environments did not differentially induce inflammation in the birds. These patterns collectively suggest that, over a period of 8 weeks, acquired immunity was more influenced by environmental microbial communities than innate immunity. Indeed, constitutive innate immunity is expected to fit evolutionary responses to different environments [15, 59], but other studies have demonstrated that innate immunity can also be flexibly adjusted to environmental differences (not specifically related to microbes) [10, 11, 13]. We did not find patterns implicating environmental microbial community features and innate immunity. This suggests certain rigor of the measured innate immune indices, at least at the time scale of this experimental study.

If the microbial environment affects animal immune function over short time scales, such as during several weeks, we expected to find changes in immune function to emerge over the course of 8 weeks of experimental treatment. Life history theory predicts that nutritional and energetic reallocation between costly immune defenses and other efforts, such as reproduction, molting, migration and thermoregulation [56, 59] invoke immunological variation between seasons or annual cycle stages [10, 11, 60]. Because such trade-offs were unlikely to be present here during 8 weeks of non-breeding under controlled ambient conditions with unlimited access to sterilized food, this could explain why our zebra finches showed no adjustment of constitutive innate immunity. Yet, we documented adjusted adaptive (IgY concentration) and induced (haptoglobin concentration) immune responses within individuals independent of treatment (Fig. 1). While these temporal shifts coincided most prominently with the radical shift from outdoor aviaries to indoor cages, both indices also showed significant increments during the experimental phase. These patterns suggest that adaptive and induced immune responses can adjust to novel microbial environments over relatively short time scales. We propose that the microbial environment may represent an important contributor to immunological variation, which should be considered in ecological immunology. Variation of immune function has been associated with variable environmental conditions in wild animals (e.g. variation imposed by long-distance migration or seasonality [10, 11, 14, 61, 62]). Our results suggest that such effects could be (partially) due to variable environmental microbial conditions, in addition to well-documented factors driving nutritional and energetic tradeoffs.

In addition to these phenotypically plastic immune responses to changing microbial environments, our evidence for significant repeatability of immune indices, within the context of the imposed experimental conditions, indicates that immunity is a characteristic property of an individual (Table 2). If this individuality has a heritable component, it may be of importance for microevolution to changing (microbial) environments [15, 43]. Devising host selection lines on different microbial conditions, and subsequent testing whether immune function upon exposure to high and low diversity microbial environments is different between animals of different lineages could greatly advance our understanding of the role of environmental microbes on evolution of animal immune systems.

Experimental microbial environments also impacted the richness, composition and stability of the cloacal microbiota of zebra finches (Figs. 2 and 3). Our detection of more OTUs in the microbiota of birds on high diversity soil, and experimental effects on beta diversity suggest that environmental bacteria shaped the host-associated microbiota and highlight that animal microbiota to some extent may reflect the microbial environment that its host experiences. Furthermore, this suggests that invasion and recruitment of environmental microbes into the animal microbiota was not fully counteracted by the host’s regulatory systems during 8 weeks of exposure. We note that our sequence data were inadequate to capture the full cloacal microbiota diversity. This likely underestimated the true effect of environmental microbes on host microbiota since less dominant taxa were likely harder to detect. Despite that caveat, our data provides further support a role of environment on host-associated microbiota, which has become increasingly recognized [31, 63–65], and sheds new light on the rarely addressed direct relationship between environmental microbes and microbiota of terrestrial vertebrates.

Nonetheless, several other studies suggested that animals also regulate their microbiota and implied importance of host genetic factors, e.g., [38, 66]. We previously reported finding no interspecific differences in cloacal, skin and feather microbiota of sympatric passerine species, and weak associations between cloacal and nest-environmental communities at the individual level [31]. This suggested importance of a shared metacommunity but also some extent of host regulation. In the current study, the pattern that zebra finch microbiota seemed to return into the direction of their baseline state also suggests that environmental bacteria might be transient rather than establishing in the cloacal microbiota over a period of 8 weeks, potentially due to host regulation. Moreover, the significance of host factors in shaping host-associated microbiota is also reflected by significant repeatability of host-associated microbiota characteristics. However, the compositional differences remained after 8 weeks of experimental treatment and longer time series are thus required to determine if host-associated microbiota remain distinct over longer periods. Collectively, these results illuminate the presence and simultaneous influences of hosts intrinsic factors and environmental microbes on animal microbiota structure but leave open whether the microbial environment also influences the ability of hosts to regulate its microbiota. Recent work on healthy humans showed for the first time evidence for a mechanistic pathway linking microbiota and adaptive immunity [47]. Systemic IgG repertoires are produced in response to various symbiotic gut commensals. The authors further postulate a protective role for anticommensal IgGs, and IgG production appeared microbiota diversity dependent as well. This evidence suggests a potential underlying mechanism for microbiota-driven adaptive immune investment. Whether such connections between microbiota and IgG (and avian IgY) production are universal across vertebrates remains to be studied. Yet, whether such antibody responses to gut microbiota can be shaped by the microbial environment should remain a topic of investigation.

Effects of environmental microbial communities on animal gut microbiota dynamics, as shown here (Fig. 3), have to our knowledge not been documented before [33]. Specifically, host-associated microbiota stabilized sooner in less diverse environments, indicating direct influence of the microbial environment on host-associated microbiota dynamics. This could be due to the differences in the taxonomic breadth of environmental microbial communities between the treatments in which case, when assuming no dispersal limitations, more diverse communities (high diversity treatment) may lead to more diverse immigration and hence increased stochasticity and longer turnover rates in host-associated microbiota (i.e. reduced resilience) [67, 68]. A fruitful avenue to test this could be to expose individual animals repetitively to a random sequence of high or low diversity microbial environments, with equal acclimation periods and simultaneous longitudinal monitoring to quantify microbiota resilience after each particular environmental transition.

Immune function significantly correlated with bacterial taxon occurrence in host-associated microbiota (Figs. 4 and 5), suggesting that immune defenses respond to host-associated microbes, or vice versa, and most dependent on occurrence rather than abundance of taxa. While immune systems have evolved to cope with microbes and other antigenic compounds, our results suggest that individuals may flexibly respond immunologically to regulate their own microbiota (Fig. 4). Since birds were translocated from group living in outdoor aviaries to indoor cages in pairs, inevitably, changes toward a sterilized diet, a different temperature regime, and altered social and microbial environments all likely contributed to the observed shift between sampling moment 1 and 2. Because of the correlative nature of these findings, experimental manipulation of immunocompetence and host-associated microbiota are necessary to establish causal relations underlying the observed association. Yet, the correlation supports results from a field study that showed links between immune function and bird-associated culturable bacterial load, but not to airborne bacterial load [62]. Although we did not explicitly consider bacterial load (total soil bacterial counts did not differ between experimental treatments, unpublished data), which has been shown previously to relate to fitness in birds [69], this work documented an individual-level relationship between immune function and host-associated microbiota while simultaneously controlling for differences in diet and other environmental microbial factors.

## Conclusions

We show that antibody-mediated immunity and the composition, richness, and dynamics of the cloacal microbiota in zebra finches varied in response to experimental microbial environments. The lack of associations between single immune indices and single host microbiota alpha-diversity measurements combined with the correlated multivariate summaries of the immune system and the microbiota underscore the complexity inherent in these systems and emphasize the challenge of interpreting immune function variation at different levels in eco-evolutionary contexts (reviewed in [15]). Yet, in a broader perspective, links between a host’s immune system and microbiota highlight the importance of incorporating microbiota analyses into studies of ecological immunology. Doing so is expected, at least partially, to provide evidence about the immunogenic agents in an organism’s environment with which an immune system must cope [15, 19, 59]. Consequently, we strongly encourage further experimental studies of the direct relationships between environmental and host-associated microbiota (e.g., [40, 70]). Ecological immunology may benefit from future investigations covering a wide range of animals, particularly when accompanied by measures of fitness. Such efforts, though challenging, are expected to make major contributions to a more mechanistic understanding of host-associated microbiota community dynamics and the microbiota’s influence on health of wild animals.

## Methods

### Experimental soils

We divided 2.5 m^3^ soil in two equal fractions and applied 3 cycles of 25 kGy gamma irradiation (Synergy Health Ede B. V, the Netherlands) to one fraction to generate a highly reduced microbial environment (‘low diversity’ soil; Additional file 1: Fig. 1). The remaining fraction constituted a high diversity microbial environment (‘high diversity’ soil). We applied in all cages either low or high diversity soil as a ~ 2-cm deep bedding layer, which we replaced every 2 weeks (mean ± SEM: 15 ± 1 days, *n* = 4). High diversity soils were stored at 4 °C enabling soil respiration while limiting bacterial activity to reduce temporal variation. Low diversity soils remained sealed and were stored under outdoor storage conditions: mean (± SEM) of 4.7 ± 0.41 °C. We maintained soil moisture content by spraying ~ 30 ml autoclaved water per cage per day. We monitored the temporal stability of soil communities by sampling soils every 3rd (*n* = 20), 10th (n = 20) and 14th (*n* = 18) day after soil was (re) placed in the cages. Soil samples were stored immediately at − 20 °C. Nine additional samples (high diversity *n* = 5, low diversity n = 4) were collected from stored bags to monitor changes during storage. A detailed description is provided in Additional file 1.

### Zebra finch husbandry

Experiments were approved by the Animal Experimentation committee of the University of Groningen (license DEC61314A), in accordance with the Dutch Law on Animal Experimentation, and standard protocols. Indoor ambient temperature was kept constant at 20 °C ± 1, relative humidity at 55% ± 15 with a 12:12 h light-dark (L:D) cycle. In the current experiment we restricted ourselves to sampling of females for practical considerations regarding sampling schemes (see Additional file 1: Table 1 for a summary of collected samples per female). Details on handling, sample processing and storage are provided as Additional file 1.

### Laboratory analysis of immune function

Non-specific antibody titers and complement-like lytic activity of blood plasma was assessed using the hemolysis-hemagglutination assay and rabbit erythrocyte antigens (Envigo, Leicester, UK) [51]. Total plasma IgY concentration was quantified in duplicate using enzyme-linked immunosorbent assays (ELISAs) with rabbit anti-chicken IgG antigens (Sigma-Aldrich, St Louis, MO, USA) (adjusted from 46, 47; detailed protocol is provided as Additional file 1*)*. Haptoglobin concentration was quantified using a commercial haem-binding assay (Tri-delta Diagnostics Inc., Morris Plains, NJ, USA) [44].

### DNA extraction, 16S rRNA gene sequencing

DNA was extracted from 250 mg of homogenized soil samples and cloacal swabs. Swab fibers were aseptically peeled from swab stalks, placed in MoBio PowerSoil DNA extraction vials (MoBio laboratories, Carlsbad, CA, USA) and DNA was isolated following the manufacturer’s protocol with addition of 0.25 g of 0.1 mm zirconia beads (BioSpec Products, Bartlesville, OK, USA) to improve cell disruption during 3 cycles of 60 s bead beating (Mini-bead beater, BioSpec Products, Bartlesville, OK, USA). Samples were characterized by (triplicate) PCR of 16S rRNA gene (V4/V5) using 515F and 926R primers, library preparation of pooled triplicates and 250 bp paired-end sequencing on an Illumina MiSeq (V2) at Argonne National Laboratory, IL, USA, following Earth Microbiota Project protocols (http://press.igsb.anl.gov/earthmicrobiota/protocols-and-standards/16s/) [71]. Seven no-sample technical negative controls for each batch of DNA extraction were included. None of the negative controls detectably produced reads in the quality-filtered sequence data set.

### Bioinformatic processing of sequence reads

Sequence reads were quality filtered and assembled using QIIME (1.9.1 [72];) retaining reads lengths ranging 368–382 bp and discarding reads (~ 267 bp) identified as zebra finch 12S rRNA gene (99% identity) using BLAST. A final 4.2 million high quality sequences were obtained (51% of raw data). OTUs were defined by 97% sequence identity with an open-reference strategy using UCLUST [73] and the Greengenes reference set (13.8 [74];). After removal of singletons, taxonomy was assigned to representative sequences based on the Greengenes reference set (97% identity). Representative sequences were then aligned using PyNast [75] and chimeric sequences were removed using UCHIME from the USEARCH81 toolkit [76] before construction of a phylogenetic tree using FastTree [77]. OTUs originating from Archaea, Chloroplast and Mitochondria were filtered from the data and the OTU table was offset to retain only OTUs that account for >0.001% of the total abundance. The QIIME pipeline is accessible as Additional file 2.

### Statistical analysis of immune function

Linear mixed-effects models (LMMs) to analyze immune indices included fixed effects for experimental group and sampling moment (0, 2, 4 and 8 weeks), as well as their interaction, and individual identity and replicate room as random effects. The probability of lytic activity was modelled using a generalized linear mixed-effects model (GLMM) with a logit link function and the same set of independent variables. ANOVA was then performed using *LmerTest* [78] with a two-tailed test. Distance-based redundancy analysis (db-RDA) in *vegan* [79] was used as a multivariate approach to test for immunological segregation of treatment groups. Repeatability *R* was calculated with a two-tailed test controlling for fixed effects using (G) LMM models with *rptR* package [54]. Confidence intervals for *R* were estimated by parametric bootstrapping and significance was inferred from two-tailed permutation tests. A detailed description is provided in Additional file 1.

### Statistical analysis of soil communities

To analyze bacterial community characteristics, *vegan* [79], *phyloseq* [80], and *lme4* [81] for R Statistical Software [82] were used. We rarefied soil samples to 1115 reads for alpha diversity estimation and then examined variation in OTU richness and Shannon diversity using LMMs with experimental treatment and time point (3, 10 and 14 days; categorical) as fixed predictors and replicate room as random effect in all models [83]. Treatment by time-interactions were not significant and removed before parameter estimation with REML. ANOVA was used with *lmerTest* [78] to estimate marginal effects (two-tailed), and *P*-values were adjusted for multiple comparisons using *multcomp* [84]. Variance-stabilizing transformation based on the fitted mean-variance relationship was applied to coverage-normalized counts [85] was performed on a non-rarefied OTU table of soil communities [55, 86], which was then used for PCoA based on the weighted UniFrac distance metric. We tested experimental treatment and temporal effects using unconstrained ordination and marginal effect estimation using two-tailed *adonis* and *adonis2* [87, 88], respectively, with permutations stratified by replicate room and 999 permutations. A detailed description is provided in Additional file 1.

### Statistical analysis of host-associated microbiota

Cloacal microbiota were analyzed similar to soil communities. Based on rarefaction curves of Shannon diversity (Additional file 1: Fig. 2), a minimum of ~ 1200 reads per sample was decided as sufficient to analyze within-sample diversity. The lack of plateau for OTU richness implied that rare OTUs were missed at the reached sampling depths. We therefore interpreted OTU richness as the dominant fraction of the microbiota. The OTU table was subset to retain the upper 80% of the coverage distribution (min: 1240 reads per sample, *n* = 145), as some cloacal samples had a low coverage (median: 3214, range: 52–88,999 reads per sample). Alpha diversity metrics were log-transformed to fulfil normality assumptions. LMMs were used to estimate effects of experimental treatment and sampling moment and included individual identity and replicate room as random effects. Pairwise contrasts of the experimental treatment factor at each sampling moment were calculated (two-tailed) using *phia* [89], and FDR-corrected *q*-values (critical *q*-value = 0.1) were reported. Temporal shifts were examined by calculating the difference of OTU richness and Shannon diversity between sampling moment *t*_*i*_ and *t*_*i-1*_ within each individual. LMMs were used to test (two-tailed) treatment and temporal shift effects. Beta diversity was calculated similarly to soil communities on a subset comprising the upper 90% of the coverage distribution of cloacal samples (*n* = 204; minimum coverage: 545 reads per sample). Within-individual shifts in the phylogenetic composition were calculated from the weighted UniFrac distance matrix and analyzed using LMM including bird identity and room as random effects and evaluated using post hoc contrasts. Negative binomial GLMs implemented in *DESeq2* [55] were used to identify differentially abundant taxa [86, 90] across sampling moments during the experiment. A detailed description is provided in Additional file 1.

### Statistical analysis of associations between immune function and microbiota

PCoA of a Bray-Curtis distance matrix of all immune indices and of (unweighted and weighted) UniFrac distance matrices of the cloacal microbiota were created using cmdscale function of *stats* [82]. A Procrustes superimposition was then applied to test whether immune function covaried with host-associated microbiota composition [91]. The protest function [91] was subsequently used to test (two-tailed) the significance of the Procrustean fit *M*^*2*^ with 10,000 permutations. Univariate regression (LMM) was applied to test associations between the first Procrustean axes of immune function and the microbiota, including sampling moment, individual identity and replicate room as random terms. Additional (G) LMMs were used to test relationships between each immune index and OTU richness, Shannon diversity, taxon occurrence (unweighted UniFrac; PCoA axis 1 and 2). A detailed description is provided in Additional file 1.

## Supplementary information

**Additional file 1.**

**Additional file 2.**

## Data Availability

The 16S rRNA gene sequence datasets generated and/or analysed in the current study are available in the EMBL-EBI European Nucleotide Archive under project accession numbers PRJEB30557 (cloacal libraries; https://www.ebi.ac.uk/ena/data/view/PRJEB30557) and PRJEB30563 (soil libraries; https://www.ebi.ac.uk/ena/data/view/PRJEB30563). The QIIME and R scripts are available as Additional files 2.

## Abbreviations

ANOVA: Analysis of variance
bp: Base pair
db-RDA: Distance-based Redundancy Analysis
DNA: Deoxyribonucleic acid
FDR: False discovery rate
ELISA: Enzyme-linked immunosorbent assay
(G)LMM: (General) linear mixed model
ID: Identity
IgM: Immunoglobulin M
IgG: Immunoglobulin G
IgY: Immunoglobulin Y
kGy: kiloGray
OTU: Operational taxonomic unit
PCoA: Principal coordinates analysis
PCR: Polymerase chain reaction
PERMANOVA: Permutational multivariate analysis of variance
phia: Post-hoc interaction analysis
QIIME: Quantitative insights into microbial ecology
REML: Restricted estimation maximum likelihood
rRNA: Ribosomal ribonucleic acid
SE: Standard error
SEM: Standard error of the mean
