# Supplementary material for: Microbial environment shapes immune function and cloacal microbiota dynamics in zebra finches Taeniopygia guttata
Source: Anim Microbiome. 2020 Jun 3;2:21. doi: 10.1186/s42523-020-00039-3 (PMC7807698; doi:10.1186/s42523-020-00039-3)
Supplement: Supplementary file 1 — Additional file 1. [file 42523_2020_39_MOESM1_ESM.zip › Additional_file_1_suppl_info_AMIC-D19-00048_R2.docx]

**Additional file 1**

This additional information accompanies:

**Microbial environment shapes immune function and cloacal microbiota dynamics in zebra finches Taeniopygia guttata**

H. Pieter J. van Veelen^1, 2^ *, Joana Falcão Salles^1^, Kevin D. Matson^3^, Marco van der Velde^1^, B. Irene Tieleman^1^

^1^ Groningen Institute for Evolutionary Life Sciences, University of Groningen, P.O. box 11103, 9700 CC, Groningen, The Netherlands.

^2^ Wetsus, European Centre of Excellence for Sustainable Water Technology, Oostergoweg 9, 9811 MA, Leeuwarden, The Netherlands.

^3^ Resource Ecology Group, Department of Environmental Sciences, Wageningen University, P.O. box 47, 6700 AA, Wageningen, The Netherlands.

* Corresponding author:

H. Pieter J. van Veelen

Wetsus, European Centre of Excellence for Sustainable Water Technology,

Oostergoweg 9, 9811 MA, Leeuwarden,

The Netherlands

Email: [pietervanveelen2@gmail.com](mailto:pietervanveelen2@gmail.com)

**Additional Methods**

*Generation, maintenance and monitoring of experimental soil beddings*

We commercially acquired (Loonbedrijf Ritzema, Zuidwolde, NL) 2.5 m^3^ soil containing clay (~40%), sand (~40%) and organic matter (~20%). We then divided the soil in two fractions and applied three cycles of 25 kGy gamma irradiation (Synergy Health Ede B.V, the Netherlands) to one soil fraction to generate a highly reduced microbial environment, referred to as ‘low diversity’ soil treatment in the main text (Fig. S1). We used the remaining fraction of non-irradiated soil as a high diversity microbial environment and referred to this fraction as ‘high diversity’ soil treatment. We applied either high or low diversity soil as a ~2-cm deep bedding layer in the cages. Biweekly, throughout the experiment, we thoroughly cleaned bedding trays and then sterilised them with 70% ethanol, followed by replacement soils (mean ± SEM: 15 ± 1 days, n = 4). Low diversity soil stocks were stored in a double layer of sealed polyethylene (PE) bags (~10 kg per bag) under ambient conditions: (mean ± SEM) 4.7 ± 0.41 ˚C (<https://weerstatistieken.nl/eelde/>). High diversity soils were stored in two layers of PE open-top bags, stored in a cooling cell at 4 ˚C enabling soil respiration while limiting bacterial activity to reduce temporal variations. We maintained soil moisture content of the cage beddings by daily spraying ~30 ml autoclaved water per cage (i.e. average daily water loss determined as weight loss over 24 hours in the experimental rooms). To monitor the temporal stability of soil bacterial communities, we sampled soils at three sampling moments between soil replacements as follows: every 3 (n=20), 10 (n=20) and 14 (n=18) days after soil (re)placement, we filled a sterile 15-ml tube with soil from three randomly selected cages in equal proportions using a sterilised spoon. Soil samples were then stored immediately at -20 ⁰C. In addition, nine samples (natural n = 5, sterilised n = 4) were taken from storage bags to monitor changes during storage.

*Housing and sample collection of zebra finches*

To experimentally test gut microbiome responses to different microbial environments, we brought 53 adult female and 54 male zebra finches *Taeniopygia guttata* from single-sex outdoor aviaries to indoor cages (50 ⨉ 50⨉ 40 cm) on 16 November 2014, housed as single-sex pairs to prevent inducing breeding behaviour. The group of females hatched between in May or September 2013 months prior to the experiment, except two birds of 3.5 years old. We exposed birds to high or low diversity soils, with two replicate rooms for high diversity soils and low diversity soils, respectively. In each room, we placed 12 cages in a block of 3 ⨉ 4, each containing a single-sex pair, each individual randomly assigned to a room and to a cage within the room. Male and female cages were spatially alternated in a block of 3 ⨉ 4 cages in each room. In addition, each room contained two separate cages to house single-sex groups of spare birds (n=0-3). We kept birds for eight weeks under the following conditions: ambient temperature at 20 ⁰C ± 1, relative humidity at 55% ± 15 and a 12:12 h light-dark (L:D) cycle.

We applied gamma-irradiation to the seed mixture fed *ad libitum* to all birds to reduce potential confounding effects of seed-associated communities on the cloacal microbiota. In addition, we provided autoclave-sterilized water with 0.2 mm-pore filter-sterilized multivitamin / amino acid solution (final concentration 4 g l^-1^; #181161; Omni-vit, Oropharma N.V., Deinz, Belgium) to compensate potential vitamin degradation from gamma-irradiation of seed. We thoroughly cleaned water and food dispensers twice a week followed by sterilisation with 70% ethanol.

To determine ‘before experiment’ levels of innate immune function and cloacal microbiome state, we collected a blood sample (~150 μl blood (8 ml kg^-1^) from the brachial vein and a cloacal swab from each female zebra finch (see Additional Table 1) and we additionally recorded body weight, wing length and tarsus length (± 0.1 mm) as a measure of general body condition. We then tracked temporal dynamics of innate immunity and the gut microbiome in the high and low diversity soil treatments by collecting cloacal swabs after two, four and eight weeks of acclimation to experimental microbial conditions. We refrained from sampling two females with congested cloacae (female ID 4208 and 4250) at the ‘before experiment’ sampling moment. We used a new pair of nitrile gloves per room when handling experimental equipment or soils and we used new gloves to handle birds, sterilised with 70% ethanol between birds. We centrifuged blood samples 10 min at 7000 rpm to separate plasma from blood cells. We then stored plasma and cloacal samples at -20 ˚C.

*Laboratory analysis of innate immune function*

To quantify non-specific antibody titres and complement-like lytic activity of female blood plasma, we used a hemolysis-hemagglutination assay with rabbit erythrocytes antigens (Envigo, Leicester, UK) ^1^. Plasma samples were randomized across plates and plate scans were scored using a double-blind procedure (by M. Havinga and M. A. Versteegh); average scores were analysed. We quantified total immunoglobulin Y (IgY) concentration in blood plasma in duplicate with an enzyme-linked immunosorbent assay (ELISA) using rabbit anti-chicken IgG (Sigma-Aldrich, St Louis, MO, USA) based on ^2,3^, optimised for zebra finch plasma (detailed protocol in *SI* *Protocols*). We used a chicken egg yolk (diluted 1:2000 in 0.1% (m/v) milk powder in 0.05% PBS-Tween 20) as standards to assess between-plate variability and as a reference to quantify total IgY concentrations in plasma samples. We examined the haptoglobin concentration in blood plasma as a measure of inflammatory status using an haem-binding assay (Tri-delta Diagnostics Inc., Morris Plains, NJ, USA) ^4^.

*DNA extraction and 16S rRNA gene amplicon sequencing of soil and cloacal samples*

We extracted DNA from 250 mg of homogenised composite soil samples and from cloacal swabs. We aseptically peeled the cotton from their cloacal swab stalks and placed them in extraction vials provided in the MoBio PowerSoil DNA isolation kit (MoBio laboratories, Carlsbad, CA, USA). We performed DNA extractions following the manufacturer’s protocol, with addition of 0.25 g of 0.1 mm zirconia beads (BioSpec Products, Bartlesville, OK, USA) to improve cell disruption during three cycles of 60 s bead beating (Mini-bead beater, BioSpec Products, Bartlesville, OK, USA). The V4/V5 region of the 16S rRNA gene was amplified in triplicate using primers 515F ^5^ and 926R ^6^ at Argonne National Laboratory, IL, USA, following the Earth Microbiome Project protocol (<http://press.igsb.anl.gov/earthmicrobiome/protocols-and-standards/16s/)> ^7^, followed by library preparation of pooled triplicates and 2 ⨉ 250 bp paired-end sequencing using V2 chemistry on an Illumina MiSeq. In total, we included seven technical negative controls in the sequencing run: three blank Powersoil DNA extractions with PCR-grade water instead of sample, as well as one with and one without zilconia beads, and two of the latter blank extractions with addition of a sterile swab. None of the samples detectably produced reads in the quality-filtered sequence data set.

*Bioinformatic processing of 16S rRNA gene amplicon sequence data*

We processed raw Illiumina sequence reads using the QIIME pipeline (v 1.9.1; ^8^). Since samples were deliberately sequenced in random order, we did not use statistical correction for sequencing run (n=2). We demultiplexed and quality filtered and paired reads, retained reads with fragment lengths ranging from 368 to 382 bp to discard non-specific reads (~ 267 bp) that mapped (99% identity) to the zebra finch’s 12S rRNA gene using BLAST. We obtained 4.2 10^6^ (51% of total read count) high quality bacterial 16S rRNA gene sequences. Using an open-reference strategy, we clustered sequences first into OTUs using the *uclust* algorithm ^9^ at 97% identity against the Greengenes reference database (v. 13.8, ^10^), and *de novo* clustered (0.1%) reads that failed to match the reference set. We selected representative sequences per OTU, concatenated both OTU tables, removed singletons to reduce effects of sequencing error on richness estimation, and annotated the resulting OTU table with taxonomic information from the Greengenes reference set (97% identity). We then aligned representative sequences using PyNast ^11^ and identified and removed chimeric sequences using the *uchime* algorithm in the *usearch81* toolkit ^12^ followed by phylogenetic tree construction using FastTree ^13^. We filtered OTUs assigned to Archaea, chloroplast and mitochondrial sequences from the data set, and offset the OTU table to retain OTUs at > 0.001% of the total abundance to reduce table sparsity. Our adjusted QIIME pipeline is available as Additional file 3).

*Statistical analysis of innate immune function*

We build linear mixed-effects models (LMMs) for each innate immune index to analyse effects of experimental soil treatment (fixed predictor) and temporal shift between sampling moment (fixed time predictor) ^14,15^ with individual female ID and experimental replicate room as random effects, as well as the interaction between fixed predictors. Because lytic capacity of plasma was mostly unobserved, we instead modelled the probability of lytic activity using a generalized linear mixed-effects model (GLMM) with a logit link function and the same set of predictors. After checking normality and homoscedasticity assumptions, we conducted ANOVAs using *LmerTest* ^16^ and calculated the repeatability R of each measure using the (G)LMM models using the rptR package ^17^, controlling for fixed effects. Confidence intervals (95%) for *R* estimated by parametric bootstrapping and the significance inferred from permutation tests. Replicate room effects were not significant in all analyses. We noted that a significant experimental effect was observed in pre-acclimation samples (Fig. 1 B) even though birds had not yet been exposed to experiment soils. Nonetheless, the temporal increase of plasma IgY in the natural soil treatment group remained after exclusion of the five individuals designated to the natural soil treatment that showed higher ‘before experiment’ values than the highest value of birds assigned to the sterilised soil treatment. This yielded significant support for the treatment:time interaction (t = -1.99, P < 0.05), suggesting that, when ‘before experiment’ plasma IgY levels were low or moderate, a more diverse microbial environment may induce an increase in circulating natural antibodies (IgY). In addition, we employed distance-based redundancy analysis (db-RDA) in *vegan* ^18^ as a multivariate approach to test immunological segregation between treatment groups. We analysed the treatment effect for each sampling moment separately to alleviate pseudo-replication complications and to remove the temporal effects.

*Statistical analysis of diversity and composition of experimental soil communities*

We used the *phyloseq* ^19^, *vegan* ^18^ and *lme4* ^20^ packages in R ^21^ to analyse soil bacterial community characteristics. All R scripts and input tables are available online.

To evaluate the effect size and stability of gamma-irradiation on alpha and beta diversity of the bacterial communities in the soil treatments, we analysed 30 high diversity and 30 low diversity soil samples (i.e. comprising three cycles of soil replacement). We seemingly mislabelled two samples collected on the same day (Sample ID 8 and 67; 10 March 2015), which we conservatively removed from the data set for this analysis. Because the estimated total diversity of natural soils was consistently higher than sterilised soils (Fig. S1a), we rarefied all samples to 1115 reads so all soil samples could be included for analysis alpha diversity in soils. Rarefaction curves for OTU richness (and estimated total diversity; Chao1) had not saturated at 1115 reads but for Shannon diversity curves reached a plateau at that coverage. We examined variation in OTU richness and Shannon diversity using linear mixed-models (LMMs) with the following predictors: experimental treatment (‘high diversity’ and ‘low diversity’), time points among soil replacements (categorical; 3, 10 and 14 days), and replicate room as a random term in all models ^15^. Interactions were not significant and were removed before estimation of fixed effects by REML. Both response variables fulfilled assumptions of normality and variance homogeneity. We estimated the marginal effect sizes using ANOVA with *lmerTest* ^16^. Where appropriate, we report adjusted *P*-values for multiple comparisons calculated using *multcomp* ^22^

Because the library sizes of our experimental soil treatments did not differ (*t* = 0.47, df = 64.6, *P =* 0.64) and sample coverage was sufficient, we performed a variance-stabilising transformation of the full non-rarefied OTU table for soils ^23,24^. We used the transformed data table for ordination analysis of phylogenetic beta diversity using the weighted UniFrac metric, visualised by principal coordinate analysis (PCoA). We tested experimental treatment and temporal effects on unconstrained ordination using tests of marginal effects with the *adonis* and *adonis2* functions, with permutations stratified by replicate room. We tested the ordinations for multivariate group dispersions between all groups using *betadisper* and *permutest* with 999 permutations.

*Statistical analysis of diversity, composition and taxon abundances of cloacal microbiomes*

We performed analysis of cloacal microbiomes using a similar approach as described for soil communities. All R scripts and input tables are available online. Based on visual evaluation of sample-wise rarefaction curves (Fig. S11), we estimated that a minimum of ~1200 reads sample^-1^ are sufficient to analyse within-sample diversity (OTU richness and Shannon diversity). Because some cloacal samples in our data set appeared to have a low coverage (median: 3398, range: 12-88999 reads per sample), we subset our OTU table to retain the upper 80% of the coverage distribution (min: 1273 reads per sample; n = 181), and rarefied the data set to the new minimum to examine variation of alpha diversity among experimental groups. We log-transformed OTU richness and Shannon’s diversity index of cloacal samples to fulfil normality and homogeneity of variance assumptions. We used LMMs to estimate effects of soil treatment and sampling moment (1, 2, 3 and 4; representing 0, 2, 4 and 8 weeks) on alpha diversity with female identity and replicate room as random effects and an interaction of the fixed effects. To test effects of experimental soil treatment on cloacal microbiome alpha diversity at each sampling moment, we calculated pairwise contrasts of the experimental treatment factor at each sampling moment using the *phia* package ^25^ and reported FDR-corrected *q*-values (critical *q*-value = 0.1). We calculated the repeatability *R* of each measure using (G)LMM using the rptR package ^17^ while controlling for fixed effects, and we estimated confidence intervals for *R* by parametric bootstrapping with significance inferred from permutation tests.

We also examined the degree of change between the sampling moments to evaluate whether cloacal microbiomes stabilised in the novel microbial environments, and if so, how soon stabilisation was achieved. To characterise the degree of change for each female, we calculated the change in OTU richness and Shannon diversity by subtracting the alpha diversity at time *t_i_* from *t_i-1_*, referred to as within-individual change. We used LMMs with the interaction between experimental treatment and time, and including replicate room as random term, to test whether the within-individual change differed between soil treatments and varied across sampling moments.

To examine beta diversity, we applied the approach described for soil beta diversity to a subset of the female cloacal microbiomes comprising the upper 90% of the coverage distribution of cloacal samples (n = 204; minimum coverage: 469 reads sample^-1^). Cloacal microbiome library sizes differed by a factor of 1.6 between treatments (*t* = 2.10, df = 129.2, *P <* 0.05), which is within acceptable range ^26^. Accordingly, we continued to examine variation in beta diversity using variance-stabilised non-rarefied data and the weighted UniFrac as a measure for phylogenetic composition. We used *adonis* to calculate the explained variance portion for experimental treatment and sampling moment, and we used *adonis2* to determine the significance of the marginal effects. Permutations were stratified by replicate room in each model.

We used the weighted UniFrac distance matrix to examine within-individual shifts in the phylogenetic composition of cloacal communities: we extracted the within-individual distances of consecutive sampling moments, and we used an LMM with a random female identity term to test whether weighted UniFrac distances varied by soil treatment, differed between sampling moments, and whether within-individual temporal shifts differed by soil treatment, i.e. an interaction term. We used Tukey-Kramer tests to evaluate post hoc group contrasts.

To examine differential abundances in cloacal microbiomes between soil treatments and with experimental duration, we used negative binomial generalised linear models as implemented in the *DESeq* function from the *DESeq2* package ^23^ to appropriately deal with overdispersed taxon abundance data ^24,27^. We used the variance-stabilised non-rarefied data set (coverage: upper 90% of samples; n = 204) for this analysis. For this analysis, we selected only the sampling moments that took place during the experiment (i.e. no baseline samples). The models included experimental treatment, experimental duration (as continuous predictor) with their interaction to predict differential taxon abundances. We reported effect sizes as log2 fold (log2FC) changes and ascribed significance using the Wald statistic (ratio of log2FC and SE): with a critical FDR-corrected *q* of 0.1 ^23^.

*The relation between immune function and cloacal microbiome characteristics*

We first generated dissimilarity-based PCoA ordinations (cmdscale function of *stats* package ^21^) of the four immune indices and unweighted UniFrac distance matrices of the cloacal microbiome, for each experimental treatment group separately. We then applied Procrustes superimposition to test whether female innate immune function covaried with cloacal microbiome composition ^28^. The employed symmetric Procrustes rotation minimizes the sum of squared distances between associated sample pairs (i.e. between simultaneously sampled blood plasma and cloacal swabs). To tests the significance of the Procrustean fit, we used the protest function ^28^, which generates a *P*-value by comparing the observed goodness-of-fit statistic *M^2^* (i.e. sum of squared residual distances) of the Procrustes rotation with a simulated *M^2^* distribution generated by recalculating *M^2^* after each of 10 000 permutations of sample labels in our empirical data. We used LMMs to test the association between the first Procrustean axes of immune function and microbiome, with sampling moment, female identity and replicate room included as random terms. Predicted ordination scores were plotted using the *effects* package ^29^. Additionally, we used corresponding (G)LMMs to explore relationships between each distinct innate immune measure and OTU richness, Shannon diversity (alpha diversity), as well as PCoA axis 1 and 2 (beta diversity) based on an unweighted UniFrac distance matrix of the female cloacal microbiome. We calculated the repeatability *R* of the first PCo axes of immune function, and the cloacal bacterial beta diversity using the (G)LMM, while controlling for fixed effects, and confidence intervals for *R* estimated by parametric bootstrapping and the significance inferred from permutation tests.

REFERENCES

1. Matson, K. D., Ricklefs, R. E. & Klasing, K. C. A hemolysis – hemagglutination assay for characterizing constitutive innate humoral immunity in wild and domestic birds. *Dev. Comp. Immunol.* **29,** 275–286 (2005).

2. Grindstaff, J. L., Demas, G. E. & Ketterson, E. D. Diet quality affects egg size and number but does not reduce maternal antibody transmission in Japanese quail Coturnix japonica. *J. Anim. Ecol.* **74,** 1051–1058 (2005).

3. Demas, G. E. & Nelson, R. J. Photoperiod and temperature interact to affect immune parameters in adult male deer mice (Peromyscus maniculatus). *J. Biol. Rhythms* **11,** 94–102 (1996).

4. Matson, K. D., Horrocks, N. P. C., Versteegh, M. A. & Tieleman, B. I. Baseline haptoglobin concentrations are repeatable and predictive of certain aspects of a subsequent experimentally-induced inflammatory response. *Comp. Biochem. Physiol. Part A* **162,** 7–15 (2012).

5. Caporaso, J. G. *et al.* Global patterns of 16S rRNA diversity at a depth of millions of sequences per sample. *Proc. Natl. Acad. Sci. U. S. A.* **108 Suppl,** 4516–22 (2011).

6. Parada, A. E., Needham, D. M. & Fuhrman, J. A. Every base matters : assessing small subunit rRNA primers for marine microbiomes with mock communities , time series and global field samples. *Environ. Microbiol.* **18,** 1403–1414 (2016).

7. Gilbert, J. A. *et al.* The Earth Microbiome Project : Meeting report of the “1st EMP meeting on sample selection and acquisition” at Argonne National Laboratory October 6 th 2010. *Stand. Genomic Sci.* **3,** 249–253 (2010).

8. Caporaso, J. G. *et al.* QIIME allows analysis of high-throughput community sequencing data. *Nat. Methods* **7,** 335–336 (2010).

9. Edgar, R. C. Search and clustering orders of magnitude faster than BLAST. *Bioinformatics* **26,** 2460–2461 (2010).

10. DeSantis, T. Z. *et al.* Greengenes, a chimera-checked 16S rRNA gene database and workbench compatible with ARB. *Appl. Environ. Microbiol.* **72,** 5069–5072 (2006).

11. Caporaso, J. G. *et al.* PyNAST: A flexible tool for aligning sequences to a template alignment. *Bioinformatics* **26,** 266–267 (2010).

12. Edgar, R. C., Haas, B. J., Clemente, J. C., Quince, C. & Knight, R. UCHIME improves sensitivity and speed of chimera detection. *Bioinformatics* **27,** 2194–2200 (2011).

13. Price, M. N., Dehal, P. S. & Arkin, A. P. Fasttree: Computing large minimum evolution trees with profiles instead of a distance matrix. *Mol. Biol. Evol.* **26,** 1641–1650 (2009).

14. Pinheiro, J., Bates, D., DebRoy, S., Sarkar, D. & R Core Team. Linear and Nonlinear Mixed Effects Models. (2017).

15. Zuur, A., Leno, E. N., Walker, N., Saveliev, A. A. & Smith, G. . *Mixed effects models and extensions in ecology with R*. (Springer, 2009).

16. Kuznetsova, A., Brockhoff, B. & Christensen, H. B. lmerTest: Tests in Linear Mixed Effects Models. (2016).

17. Stoffel, M. A. & Nakagawa, S. rptR : repeatability estimation and variance decomposition by generalized linear mixed-effects models. *Methods Ecol. Evol.* **8,** 1639–1644 (2017).

18. Oksanen, J. *et al.* vegan: Community Ecology Package. (2017).

19. McMurdie, P. J. & Holmes, S. Phyloseq: An R Package for Reproducible Interactive Analysis and Graphics of Microbiome Census Data. *PLoS One* **8,** e61217 (2013).

20. Bates, D., Mächler, M., Bolker, B. & Walker, S. Fitting Linear Mixed-Effects Models Using {lme4}. *J. Stat. Softw.* **67,** 1–48 (2015).

21. R Core Team. R: A language and environment for statistical computing. (2016).

22. Hothorn, T., Bretz, F., Westfall, P. & Heiberger, R. M. Package ‘multcomp’ Title Simultaneous Inference in General Parametric Models. **50,** 346–363 (2016).

23. Love, M. I., Huber, W. & Anders, S. Moderated estimation of fold change and dispersion for RNA-seq data with DESeq2. *Genome Biol.* **15,** 550 (2014).

24. Weiss, S. *et al.* Normalization and microbial differential abundance strategies depend upon data characteristics. *Microbiome* **5,** 27 (2017).

25. De Rosario-Martinez, H. phia: Post-Hoc Interaction Analysis. (2015).

26. Weiss, S. J. *et al.* Effects of library size variance, sparsity, and compositionality on the analysis of microbiome data. *PeerJ* e1408 (2015). doi:10.7287/peerj.preprints.11

27. McMurdie, P. J. & Holmes, S. Waste Not, Want Not: Why Rarefying Microbiome Data Is Inadmissible. *PLoS Comput. Biol.* **10,** e1003531 (2014).

28. Peres-neto, P. R. & Jackson, D. A. How well do multivariate data sets match ? The advantages of a Procrustean superimposition approach over the Mantel test. *Oecologia* **129,** 169–178 (2001).

29. Fox, J. Displays in R for Generalised Linear Models. *J. Stat. Softw.* **8,** 1–27 (2003).

**Additional Protocols**

**Quantification of antibody types IgG/Y in avian blood plasma**

*Authors:*

*H. Pieter J. van Veelen^1^, Maaike A. Versteegh^1^, B. Irene Tieleman^1^*

^1^ Groningen Institute for Evolutionary Life Sciences, University of Groningen, P.O. box 11103, 9700 CC, Groningen, The Netherlands.

This protocol is based on: Grindstaff *et al.* and Demas and Nelson

**Materials:**

- Nunc immuno Maxisorp flat bottom plates (Sigma M9410; VWR 735-0083)
- Rabbit anti-chicken IgY (Sigma C6409-2ML)
- AP conjugated rabbit, antichicken IgG (Sigma A9171)
- Antichicken IgY (Promga G116A)
- Carbonate/bicarbonate buffer (Sigma C3041)
- p-Nitrophenyl phosphate buffer (Sigma N1891)
- Diethanolamine buffer (Sigma D8885)
- PBS (Sigma P4417)
- Milk powder (Milk powder, Campina).
- MgCl_2_ (Sigma M8266)

**Reagents to make:**

- Carbonate buffer (0.15 M, pH 9.6; needed 10 ml/plate): 3 capsules Carbonate in 100 ml H_2_O. adjust pH to 9.6 with HCl.
- PBS-Tw: 5 pills PBS in 1L H_2_O, add 0.5 ml Tween 20
- Diethanolamine buffer (0.1 M, pH 9.5, 5 mM MgCl_2_): 1.9 ml diethanolamine in 200 ml H_2_O, add 0.2033 gr MgCl_2_.6H_2_O. Adjust pH to 9.5 (exactly!) with HCl.
- Substrate Buffer (*prepare shortly before use!).* 0.1 M -nitrophenyl phosphate buffer in Diethanolamine buffer. Add 0.3711 gr PNPP (fridge) to 10 ml diethanolamine buffer
- Antichicken IgG solution: 3 ug Rabbit antichicken IgG in 1 mL Carbonate buffer (0.15 M, pH 9.6).
- 0.5%-Milk solution: 5 g milk powder in 1 Liter PBS + 0.5 mL Tween 20
- 0.1%-Milk solution: 1 g milk powder in 1 Liter PBS + 0.5 mL Tween 20
- Labelling antibody-solution: dilute AP conjugated rabbit, anti-chicken IgG (Sigma A9171) 1:1000 in 0.1% milk solution.
- Substrate buffer (taken from Demas and Nelson): 0.1 M Nitrophenyl phosphate buffer in 0.1 M diethanolamine buffer [pH 9.5] containing 5 mM MgCl_2_.

**Protocol**

Day 1

1. Prepare per plate 10 ml antichicken IgG solution: 6.13 µl Rabbit anti-chicken IgG (4.9 mg/ml) in 10 ml Carbonate buffer.
2. Coat the Elisa plates with 90 µl antichicken IgG solution and incubate overnight at 4 degrees Celsius.

Day 2

1. Prepare per plate 10 ml 0.5%-Milk-dilution (50 mg milkpowder per 10 ml PBS-Tween 20)
2. Empty the plates. Block the plates with 90 µl per well of 0.5%-Milk-dilution and incubate at room temperature for at least 2 hours (Marco: 4 hours worked)
3. Prepare 0.1% milk solution for Standard Curve and sample preparation.
4. Prepare Standard Curve: 100 mg chicken yolk in 0.5 ml 0.1% milk solution.
5. Prepare samples: make 1/6400 dilution of zebra finch plasma samples in 200 µl 0.1% milk solution.
6. Add 7-9 glass beads to each sample yolk sample and vortex for 45-60 min.
7. Make Diluted-SC 1/32768: 20 µl SC + 620 µl 0.1% milk solution, mix and transfer 20 µl to 620 µl 0.1% milk solution, mix and transfer 20 µl to 620 µl 0.1% milk solution (now you have 1/32768 diluted Standard Curve. Make enough for all plates! Make tube for each serial dilution.
8. After > 2h. Empty the plates, wash 1x with PBS-tw (and tap on counter on piece of paper, to empty properly).
9. Add 90 µl 0.1% milk solution to all SC wells. Not if you make separate tube for each serial dilution. (and samples if you test serial dilutions).
10. Add 90 µl diluted SC to 1^st^ well (duplo), and serial dilute (1/2x). Last well = 0.1% milk solution only = blanco. Or add 90 µl for serial diluted SC tubes.
11. Add 90 µl of each sample to wells (duplo).
12. Incubate the plate overnight at 4 degrees.

Day 3

1. Switch on the water-bath or incubator at 37˚C.
2. Prepare 10 ml 0.1% milk solution (make 25 ml for easy weighing purpose).
3. Empty and wash the plate 4-6 times with 90 µl per well PBS-tween 20. Wash 3x.
4. Prep 10 ml labelling antibody-solution: 10 µl AP conjugated rabbit anti-chicken + 10 ml 0.1% milk solution. Add 90 µl of labelling antibody-solution to each well. Incubate 1 hour at 37˚C. Put the plate as flat as possible, so that the plate is heated uniformly. (Optional: turn around after 30 minutes)
5. Set the plate-reader at 405 nm, and at kinetic read. Set it so that it reads every 30s, for 60 minutes. Set file format to time.
6. Empty and wash 3 times with 90 µl per well PBS-tween 20 (after last time: tap on counter). Make sure the bottom of the plate is ‘clean’ (i.e. no water, parafilm etc.).
7. Prep Substrate buffer: add 0.3711 gr PNPP (fridge) to 10 ml Diethanolamine buffer.
8. At plate-reader desk, add 90 µl substrate buffer in each well
9. Immediately start reading with plate-reader. Close drawer, and press read (set at 405 nm wavelength every 30s for 60 minutes).

*Determining initial sample concentration*

When aiming to assess total IgY concentrations in a novel model or non-model species for which the optimal sample dilution is unknown, we needed to start determining the initial concentration. To do this, we first made a pool of several samples to make sure that they reflect the expected variation structure (treatment, sampling moment) of the samples to be investigated. Then, we serially diluted this pooled sample. We started with a 1:100 dilution and serially diluted 1:4 with each step, 8 steps in total, i.e. 1:100; 1:400, 1:1600, 1:6400, 1: 12800; 1:25600; 1:10240000; 1:409600.

The resulting data were plotted as absorbance against the inversed sample dilution. We chose to quantify antibodies in the actual sample set using the dilution that provides the widest range of variation, i.e. the middle (linear) part of the negative slope. Based on our data we opted for a dilution between 1:25600 and 1:102400. Sample IgY levels were then determined by the absorbance at a fixed time point of 28 min after initiation of the enzymatic reaction.

**References**

Grindstaff, J. L., Demas, G. E. & Ketterson, E. D. Diet quality affects egg size and number but does not reduce maternal antibody transmission in Japanese quail Coturnix japonica. *J. Anim. Ecol.* **74,** 1051–1058 (2005).

Demas, G. E. & Nelson, R. J. Photoperiod and temperature interact to affect immune parameters in adult male deer mice (Peromyscus maniculatus). *J. Biol. Rhythms* **11,** 94–102 (1996).

**Additional Figures**

**
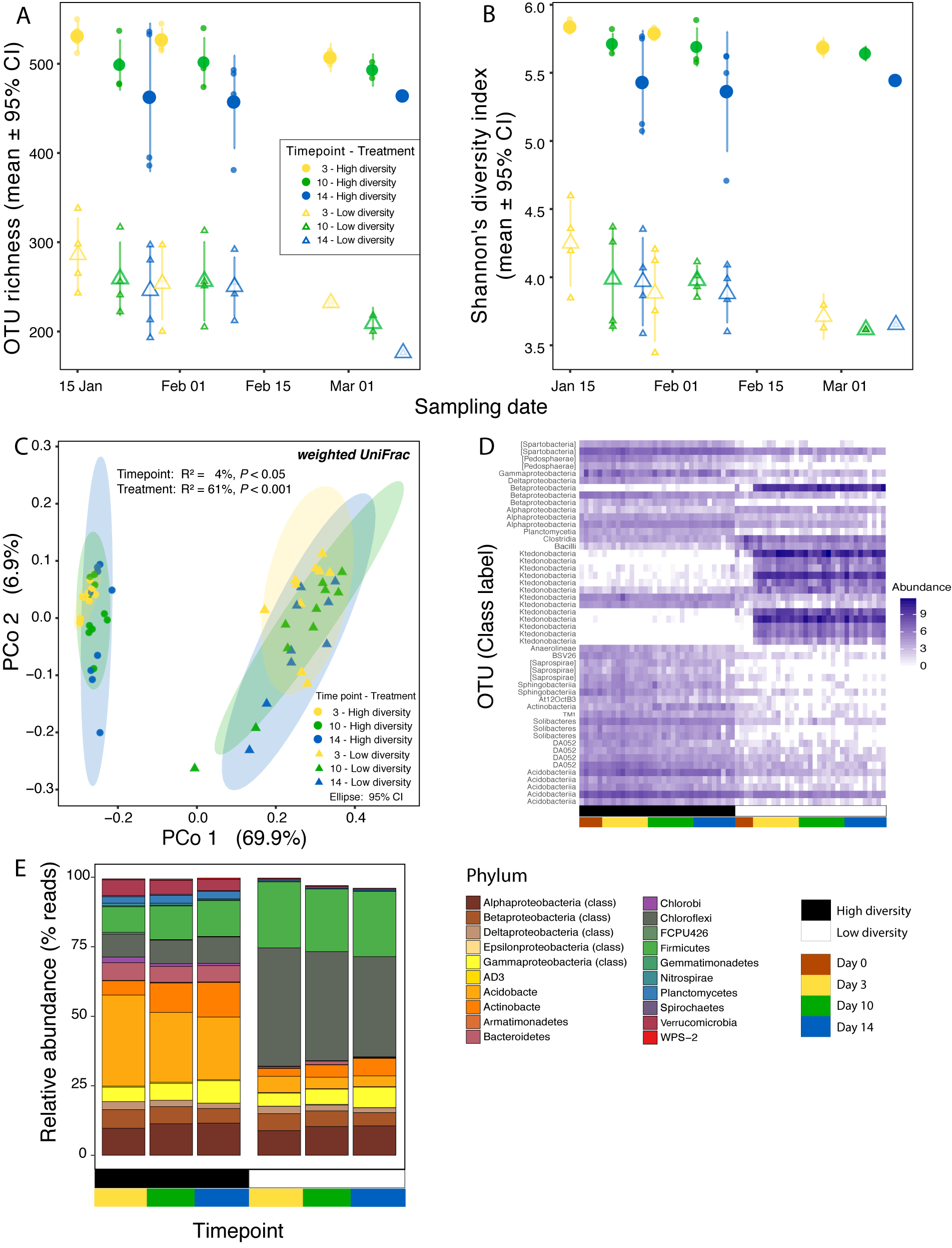
**

**Additional Figure 1. Experimental soil bacterial community characteristics.** Relationship of a) OTU richness and b) Shannon diversity with sampling date during the experiment for each experimental soil treatment (closed circle = high diversity soil, open circle = low diversity soil) across time points between soil replacements (red = 0 days, yellow = 3 days, green = 10 days, blue = 14 days). c) Principal coordinate analysis (PCoA) using weighted UniFrac distances of soil samples without ‘day 0’. d) Heatmap of soil samples showing variance-stabilised abundances of 50 most differentially abundant OTUs (labelled by bacterial class). Day 0 samples have been taken straight out of the sterilised bags. Plating Day 0 samples on TSA and LB media was negative. We conclude that the Day 0 soil microbiota samples thus largely reflect relic DNA from dead bacterial cells right after gamma irradiation. e) Bacterial community structure represented by mean relative abundances of major bacterial groups (colours), stratified by time point for each of the two soil treatments. a) LMM OTU richness ANOVA; experimental treatment: *F*_1,54_ = 551.2, *P* < 0.001; time point: *F*_2,54_ =5.58, *P* < 0.01; b) LMM Shannon diversity ANOVA; experimental treatment: *F*_1,54_ = 600.7, *P* < 0.001; time point: *F*_2,54_ =4.08, *P* < 0.05. c) PERMANOVA: experimental treatment: Pseudo-*F*_1,54_ = 96.7, *P* < 0.001; time point: Pseudo-*F*_2,54_ =3.32, *P* < 0.05.

**
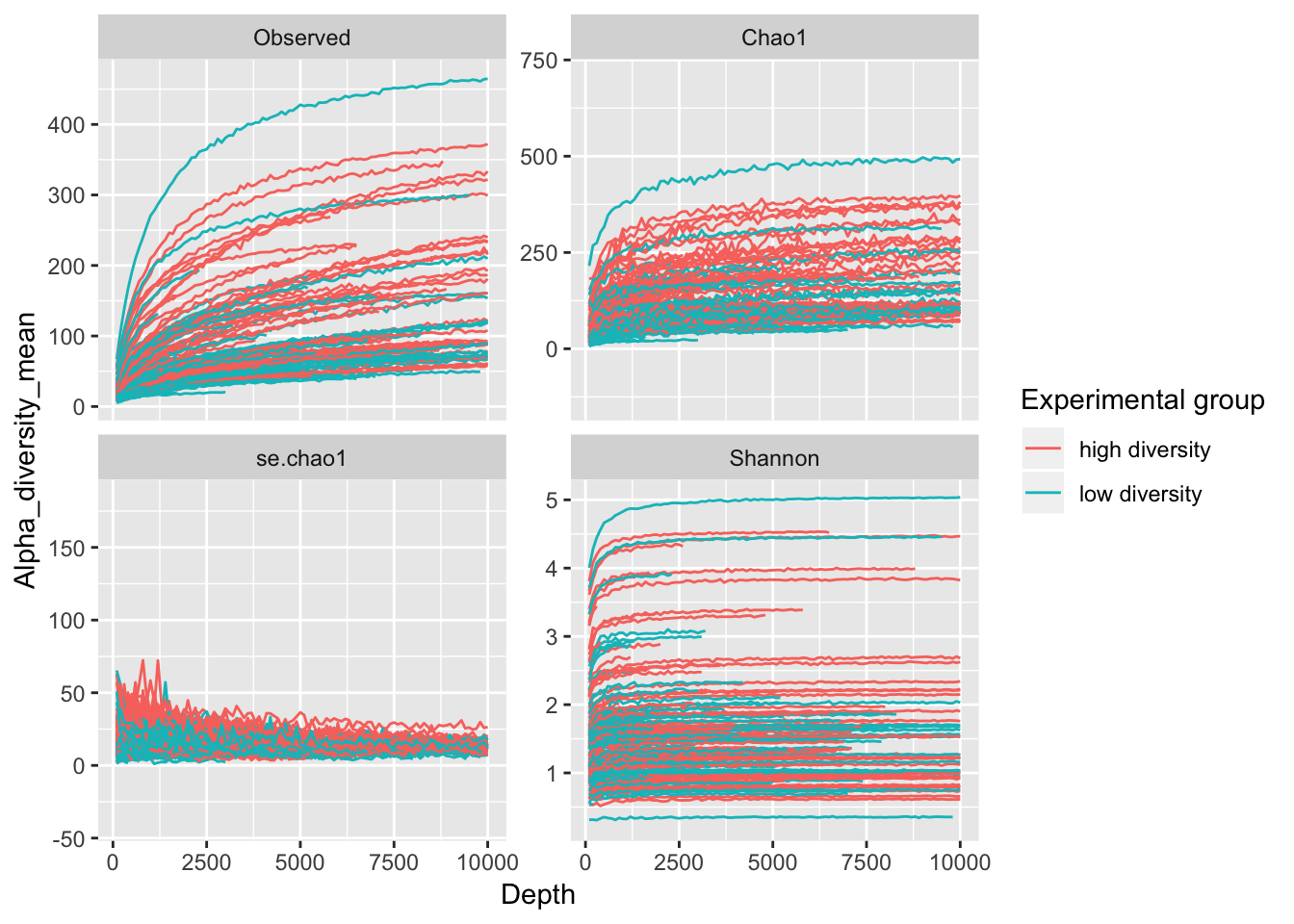
**

**Additional Figure 2. Rarefaction curves of cloacal microbiome samples.** Rarefaction curves for four alpha diversity metrics. Alpha diversity was estimated as the average value of ten random subsamplings at each depth. Shannon diversity saturated in all samples at ~1200 reads per sample.

**
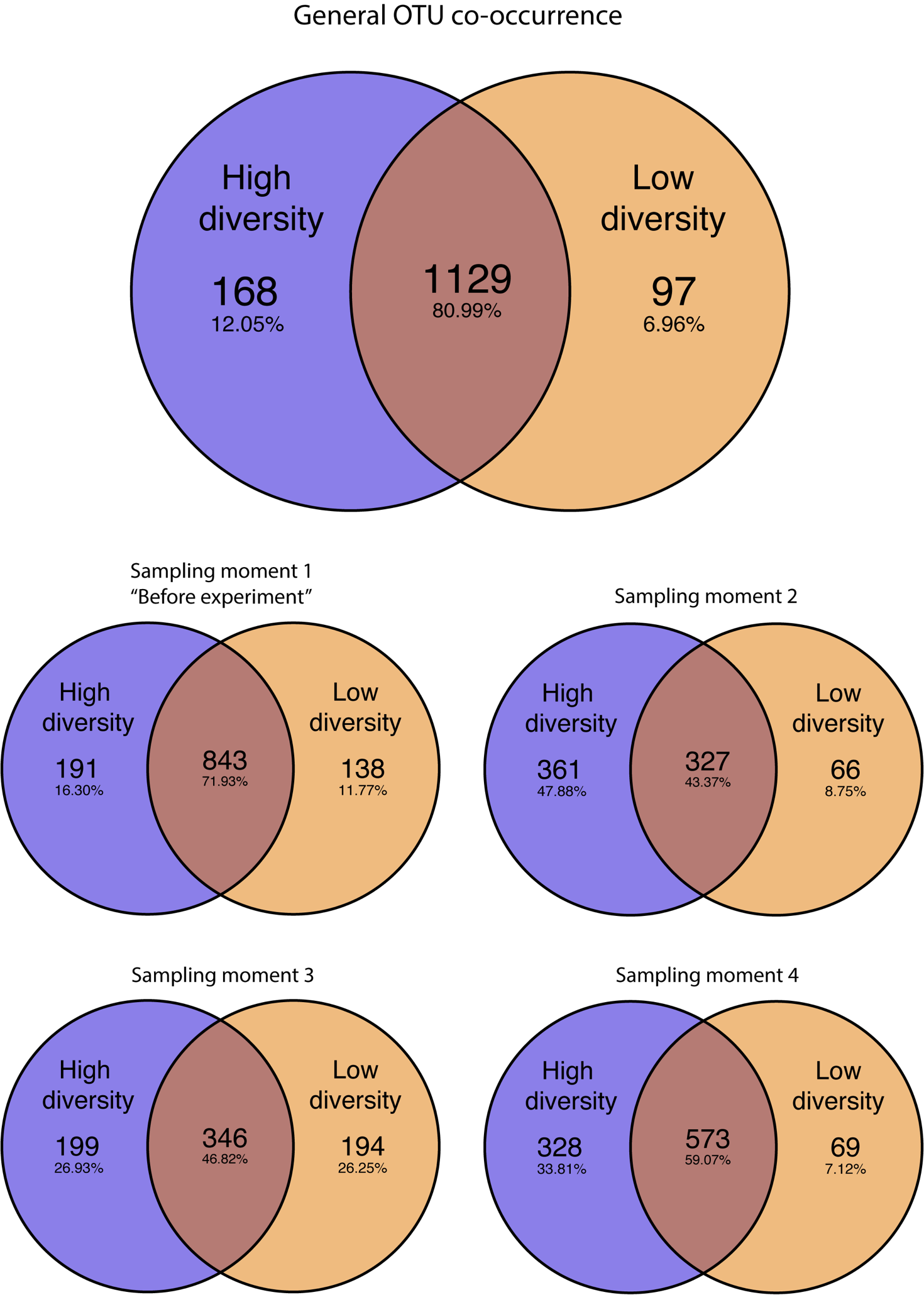
**

**Additional Figure 3. Uniqueness and co-occurrence between cloacal bacterial communities of birds on high and low diversity soils.** Values (and percentages) in each compartment depict the (relative) number of unique or overlapping OTUs identified in the cloacal microbiomes of females acclimating to either high diversity or low diversity soils. Small venn diagrams depict separate analyses per sampling moment, showing that the average relative OTU co-occurrence between soil treatments decreased after birds were exposed to distinct microbial environments. Compartments are not scaled to the number of OTUs.


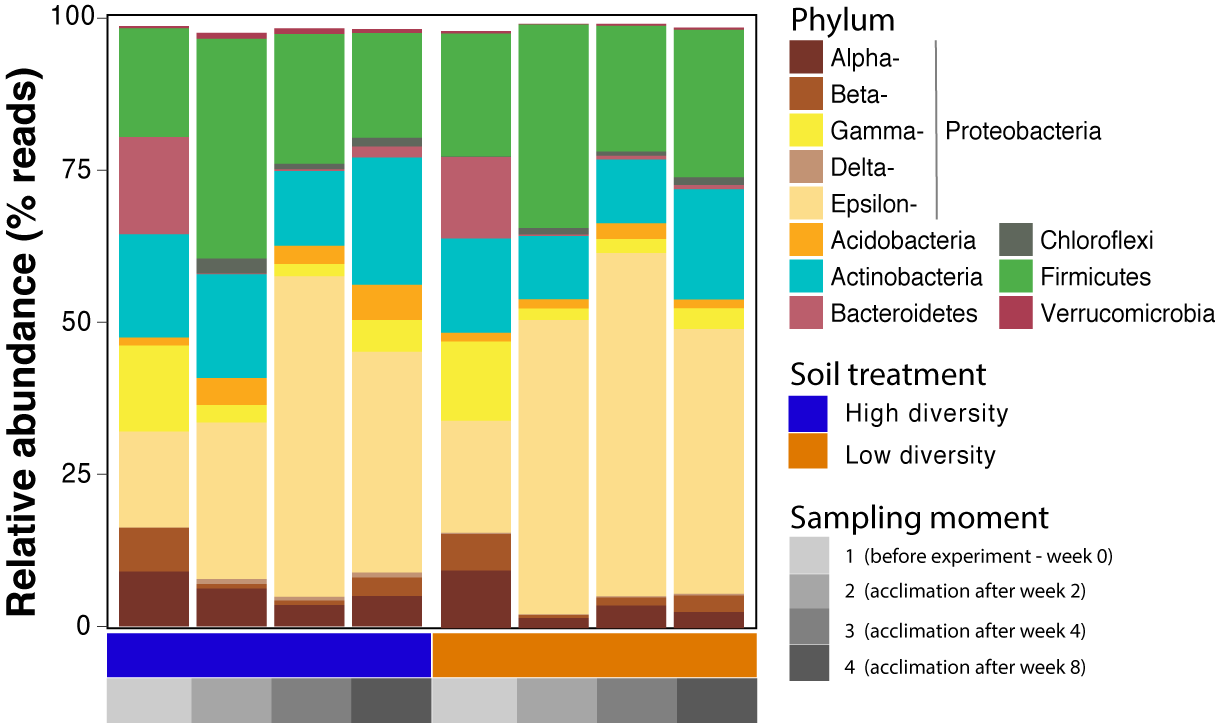


**Additional Figure 4. Relative taxon abundance depicting cloacal bacterial community structure**. Relative read abundances as proxy for bacterial taxon abundances, for which the top seven dominant phyla have been shown. The dominant bacterial phylum Proteobacteria is divided into class-level taxa. Each stacked bar shows mean relative abundances for each sampling moment and for both experimental soil treatments separately.

**
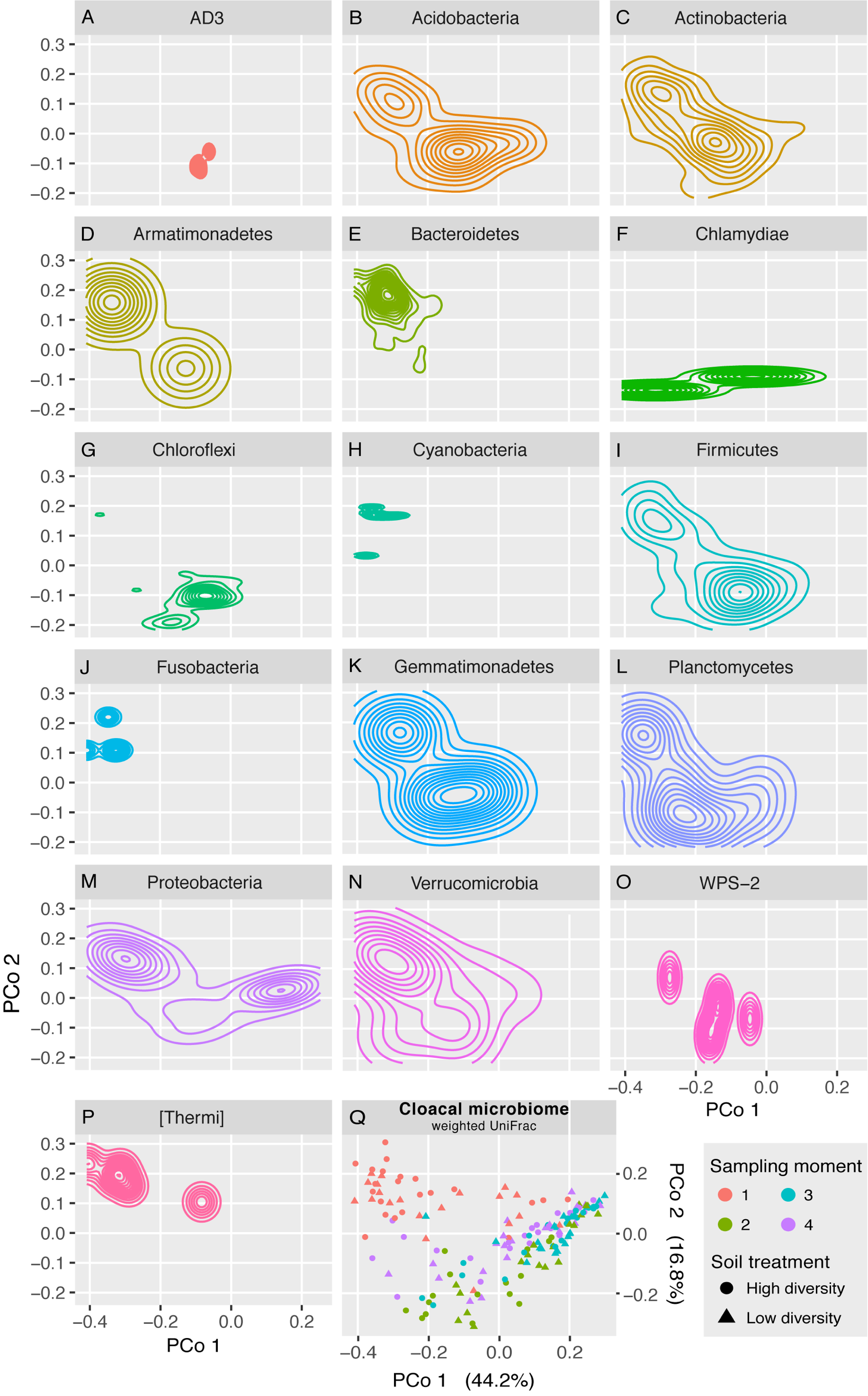
**

**Additional Figure 5. Ordination of bacterial phyla shows associations of bacterial phyla with sample ordination**. Density plots of OTUs belonging to bacterial phyla (A-P) are plotted separate from the cloacal microbiome samples (Q; Fig. 2C in main text) in PCoA ordinations of weighted UniFrac distances. Phylum-specific patterns of bacterial OTU densities show associations with temporal shifts in bacterial community composition in the cloacae of female zebra finches.


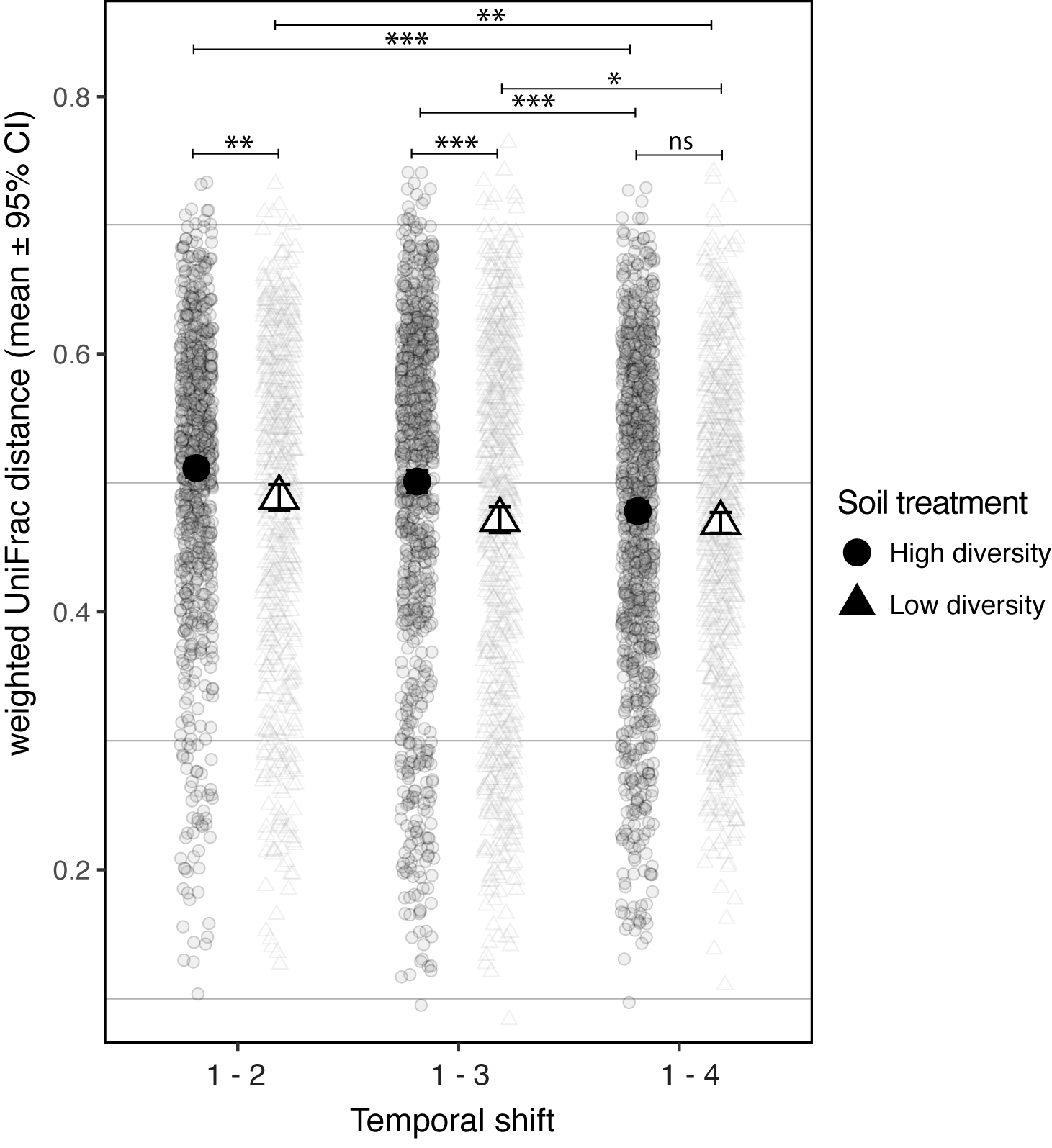


**Additional Figure 6. Pairwise weighted UniFrac distances between experimental and before-experiment states of cloacal bacterial communities.** Cloacal samples of high diversity (circles) and low diversity (triangles) treatments during the experimental sampling moments (2, 3 and 4) are compared to the before-experiment samples (1). Cloacal samples in the low diversity soil treatment are significantly more similar (lower values) to the before-experiment samples during sampling moment 2 and 3, but not 4. However, all cloacal communities (on high and low diversity soils) tend to return back to the before-experiment state, as at sampling moment 4, cloacal communities are more similar to the before-experiment state compared with sampling moments 2 and 3. * FDR *q* < 0.05, ** *q* < 0.01, *** *q* < 0.001, ns = not significant. Global test; treatment: *F*_1,5034_ = 31.3, *P* < 0.001; temporal shift: *F*_2,5034_ = 19.3, *P* < 0.001.

**
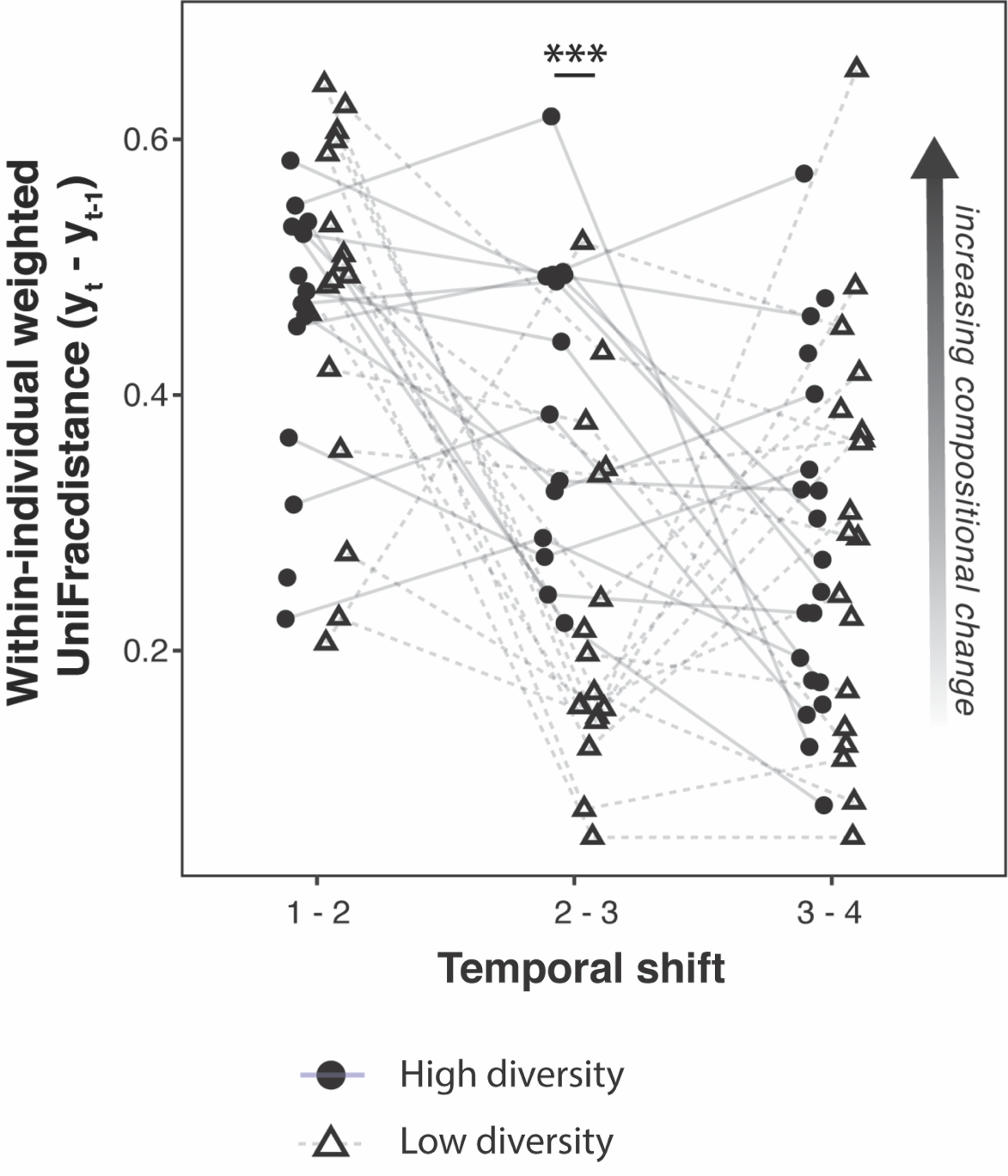
**

**Additional Figure 7. Longitudinal representation of within-individual shifts in phylogenetic community composition.** Individual data points depict within-individual weighted UniFrac distances between consecutive sampling points (sampling interval). Data point belonging to females on high diversity soil are depicted by solid circles and females on low diversity soils by open triangles. See Table 5 in the main text for statistics.

**
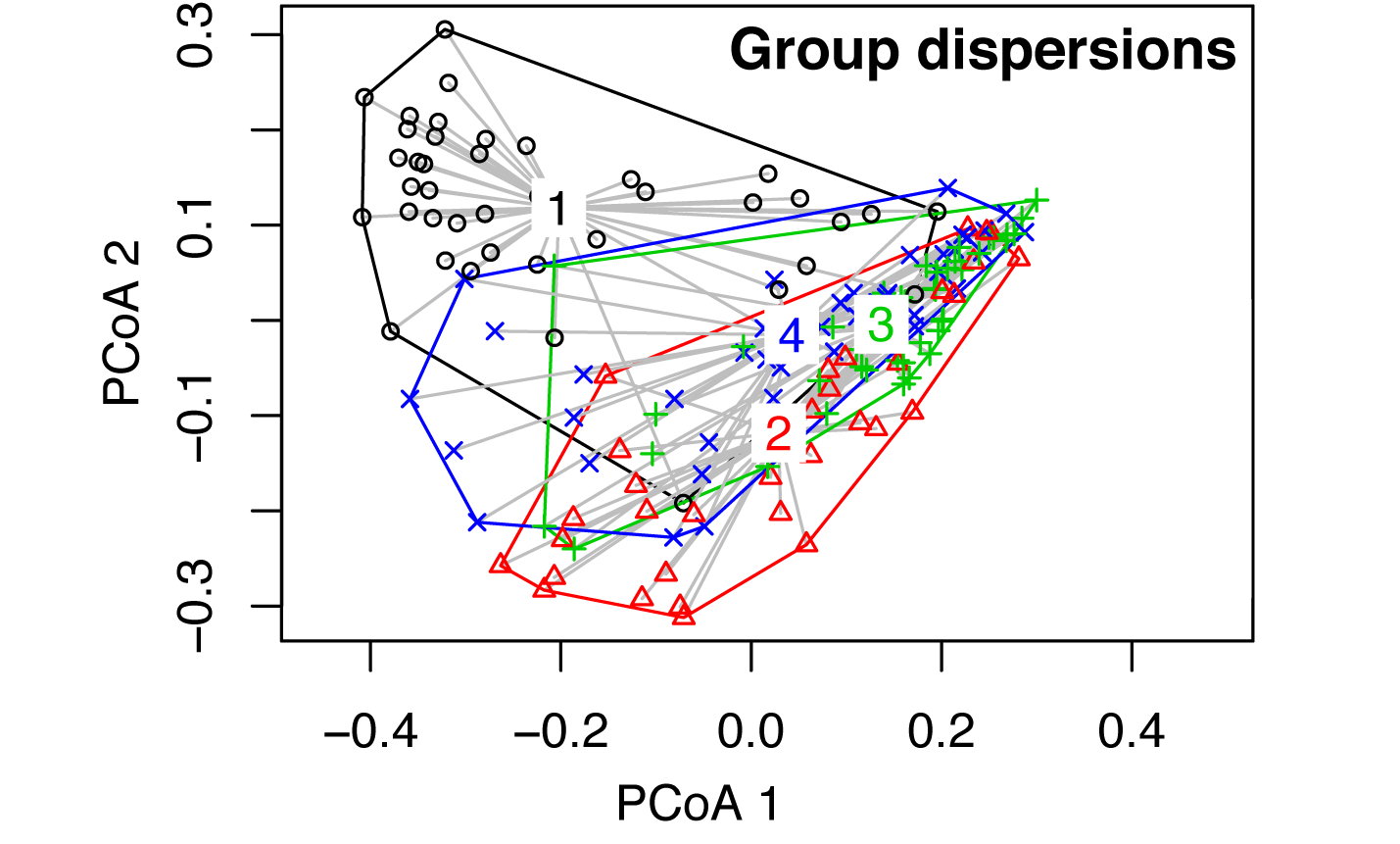
**

**Additional Figure 8. Group dispersions of PCoA clusters of cloacal microbiomes at different sampling moments during the experiment.** Group dispersions are calculated as distances to the centroid for each sampling point. PERMANOVA statistics accompanying this Figure are reported in Table 5 in the main text.

**
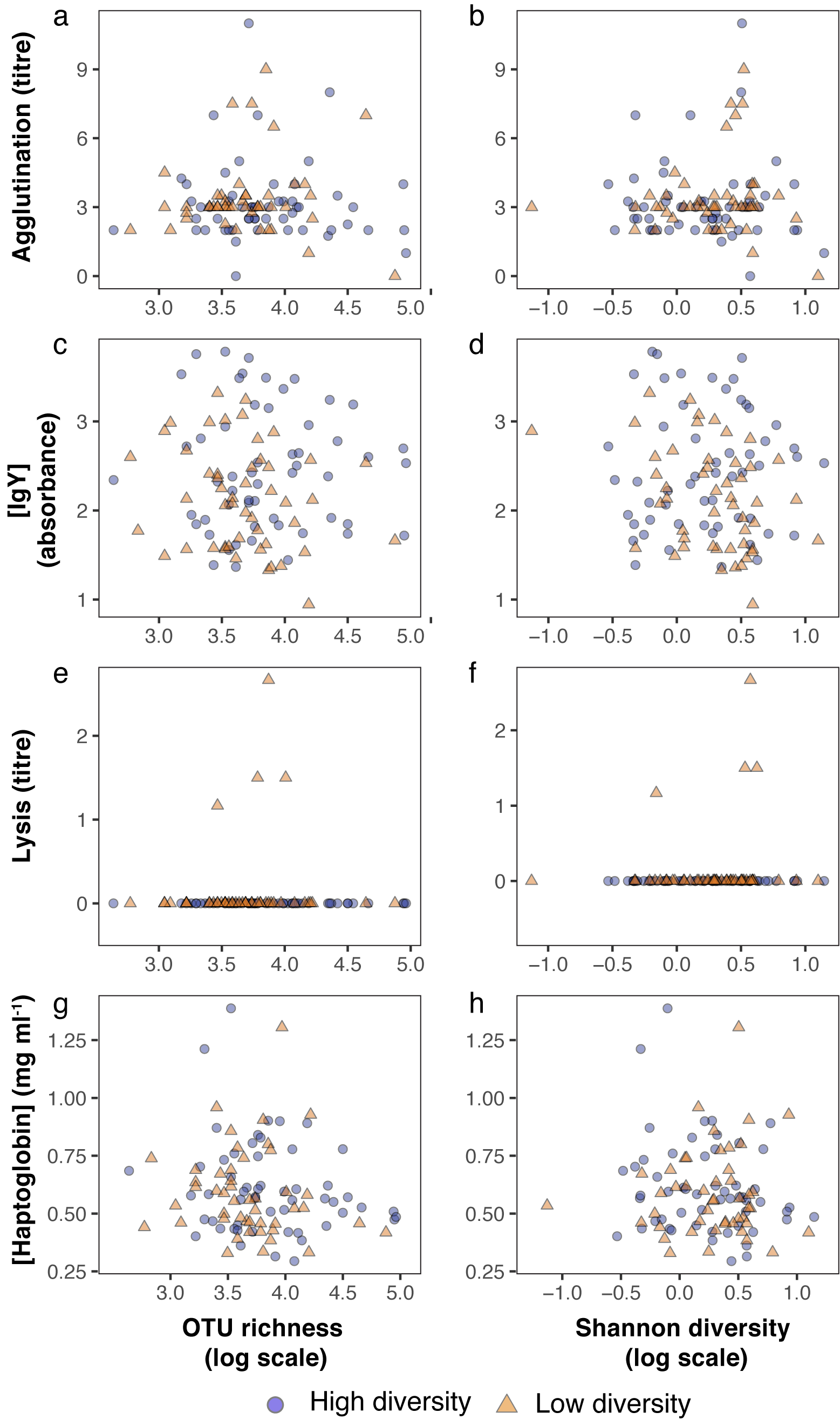
**

**Additional Figure 9. Associations between innate immune indices and alpha diversity of cloacal microbiomes.** LMMs revealed no statistical support for experimental soil treatment, experimental duration or their interaction.

**Additional tables**

**Additional Table 1**. Overview of collected cloacal gut samples of zebra finches on high diversity and low diversity soils.

**High diversity soil Low diversity soil**

**s**ampling time points sampling time points

| **Female ID** | **1** | **2** | **3** | **4** | **Total** |  | **1** | **2** | **3** | **4** | **Total** |
| --- | --- | --- | --- | --- | --- | --- | --- | --- | --- | --- | --- |
| 2744 |  |  |  |  |  |  | 1 | 1 | 1 | 1 | **4** |
| 2784 | 1 | 1 | 1 | 1 | **4** |  |  |  |  |  |  |
| 3666 | 1 | 1 | 1 | 1 | **4** |  |  |  |  |  |  |
| 3759 |  |  |  |  |  |  | 1 | 1 | 1 | 1 | **4** |
| 3804 | 1 | 1 | 1 | 1 | **4** |  |  |  |  |  |  |
| 3811 |  |  |  |  |  |  | 1 | 1 | 1 | 1 | **4** |
| 3824 |  |  |  |  |  |  | 1 | 1 | 1 | 1 | **4** |
| 3875 | 1 | 1 | 1 | 1 | **4** |  |  |  |  |  |  |
| 4205 |  |  |  |  |  |  | 1 | 1 | 1 | 1 | **4** |
| 4207 |  |  |  |  |  |  | 1 | 1 | 1 | 1 | **4** |
| 4208 |  |  |  |  |  |  |  | 1 | 1 | 1 | **3** |
| 4211 |  |  |  |  |  |  | 1 | 1 | 1 | 1 | **4** |
| 4215 |  |  |  |  |  |  | 1 | 1 | 1 | 1 | **4** |
| 4218 | 1 | 1 | 1 | 1 | **4** |  |  |  |  |  |  |
| 4227 |  |  |  |  |  |  | 1 | 1 | 1 | 1 | **4** |
| 4229 |  |  |  |  |  |  | 1 | 1 | 1 | 1 | **4** |
| 4231 | 1 | 1 | 1 | 1 | **4** |  |  |  |  |  |  |
| 4232 | 1 | 1 | 1 | 1 | **4** |  |  |  |  |  |  |
| 4237 | 1 | 1 | 1 | 1 | **4** |  |  |  |  |  |  |
| 4238 | 1 | 1 | 1 | 1 | **4** |  |  |  |  |  |  |
| 4244 | 1 | 1 | 1 | 1 | **4** |  |  |  |  |  |  |
| 4250 |  |  |  |  |  |  |  | 1 | 1 | 1 | **3** |
| 4254 | 1 | 1 | 1 | 1 | **4** |  |  |  |  |  |  |
| 4256 | 1 | 1 | 1 | 1 | **4** |  |  |  |  |  |  |
| 4271 | 1 | 1 | 1 | 1 | **4** |  |  |  |  |  |  |
| 4280 |  |  |  |  |  |  | 1 | 1 | 1 | 1 | **4** |
| 4298 | 1 | 1 | 1 | 1 | **4** |  |  |  |  |  |  |
| 4406 |  |  |  |  |  |  | 1 | 1 | 1 | 1 | **4** |
| 4414 | 1 | 1 | 1 | 1 | **4** |  |  |  |  |  |  |
| 4427 |  |  |  |  |  |  | 1 | 1 | 1 | 1 | **4** |
| 4428 | 1 | 1 | 1 | 1 | **4** |  |  |  |  |  |  |
| 4432 |  |  |  |  |  |  | 1 | 1 | 1 | 1 | **4** |
| 4434 |  |  |  |  |  |  | 1 | 1 | 1 | 1 | **4** |
| 4438 | 1 | 1 | 1 | 1 | **4** |  |  |  |  |  |  |
| 4445 | 1 | 1 | 1 | 1 | **4** |  |  |  |  |  |  |
| 4453 |  |  |  |  |  |  | 1 | 1 | 1 | 1 | **4** |
| 4454 |  |  |  |  |  |  | 1 | 1 | 1 | 1 | **4** |
| 4457 | 1 | 1 | 1 | 1 | **4** |  |  |  |  |  |  |
| 4459 | 1 | 1 | 1 | 1 | **4** |  |  |  |  |  |  |
| 4460 | 1 | 1 | 1 | 1 | **4** |  |  |  |  |  |  |
| 4464 |  |  |  |  |  |  | 1 | 1 | 1 | 1 | **4** |
| 4466 | 1 | 1 | 1 | 1 | **4** |  |  |  |  |  |  |
| 4469 |  |  |  |  |  |  | 1 | 1 | 1 | 1 | **4** |
| 4471 |  |  |  |  |  |  | 1 | 1 | 1 | 1 | **4** |
| 4665 | 1 | 1 | 1 | 1 | **4** |  |  |  |  |  |  |
| 4669 | 1 | 1 | 1 | 1 | **4** |  |  |  |  |  |  |
| **Grand Total** | **24** | **24** | **24** | **24** | **96** |  | **20** | **22** | **22** | **22** | **86** |
